# Combinations of deletion and missense variations of the dynein-2 DYNC2LI1 subunit found in skeletal ciliopathies cause ciliary defects

**DOI:** 10.1038/s41598-021-03950-0

**Published:** 2022-01-07

**Authors:** Hantian Qiu, Yuta Tsurumi, Yohei Katoh, Kazuhisa Nakayama

**Affiliations:** 1 Department of Physiological Chemistry, Graduate School of Pharmaceutical Sciences, Kyoto University, Sakyo-ku, Kyoto, 606-8501 Japan; 2 Present Address: General Research Institute, Hoyu Co., Ltd., Nagakute, Aichi 480-1136 Japan

**Keywords:** Biochemistry, Cell biology

## Abstract

Cilia play crucial roles in sensing and transducing extracellular signals. Bidirectional protein trafficking within cilia is mediated by the intraflagellar transport (IFT) machinery containing IFT-A and IFT-B complexes, with the aid of kinesin-2 and dynein-2 motors. The dynein-2 complex drives retrograde trafficking of the IFT machinery after its transportation to the ciliary tip as an IFT cargo. Mutations in genes encoding the dynein-2-specific subunits (DYNC2H1, WDR60, WDR34, DYNC2LI1, and TCTEX1D2) are known to cause skeletal ciliopathies. We here demonstrate that several pathogenic variants of DYNC2LI1 are compromised regarding their ability to interact with DYNC2H1 and WDR60. When expressed in *DYNC2LI1*-knockout cells, deletion variants of DYNC2LI1 were unable to rescue the ciliary defects of these cells, whereas missense variants, as well as wild-type DYNC2LI1, restored the normal phenotype. *DYNC2LI1*-knockout cells coexpressing one pathogenic deletion variant together with wild-type DYNC2LI1 demonstrated a normal phenotype. In striking contrast, *DYNC2LI1*-knockout cells coexpressing the deletion variant in combination with a missense variant, which mimics the situation of cells of compound heterozygous ciliopathy individuals, demonstrated ciliary defects. Thus, DYNC2LI1 deletion variants found in individuals with skeletal ciliopathies cause ciliary defects when combined with a missense variant, which expressed on its own does not cause substantial defects.

## Introduction

The movement of cargos (proteins, and membrane-bound vesicles and organelles) along microtubules, as well as the remodeling of microtubules during cell division, are coordinately controlled by motor proteins. In general, the kinesin and dynein motors drive plus- and minus-end directed movements of cargos, respectively^1^.

Cilia are microtubule-based projections from the surfaces of a variety of eukaryotic cells, and perceive and transduce mechanical signals, such as fluid flow, and biochemical signals, such as the Hedgehog (Hh) family of morphogens. To achieve these functions, specific proteins exist on the ciliary membrane, including G protein-coupled receptors (GPCRs) and ion channels^2–4^. For example, GPR161 and Smoothened (SMO) are ciliary GPCRs that regulate Hh signaling; GPR161 participates in the basal repression of Hh signaling, whereas upon Hh pathway activation, GPR161 exits cilia and SMO gains entry into cilia to stimulate events downstream of Hh^3^. Owing to the crucial roles of cilia in development and homeostasis of organisms, their dysfunction causes a heterogenous group of disorders known as the ciliopathies, which demonstrate a broad spectrum of symptoms, including skeletal and brain malformation^5,6^.

Not only bidirectional protein trafficking within cilia, but also the entry and exit of proteins across the ciliary gate composed of the transition zone (TZ) is mediated by the intraflagellar transport (IFT) machinery ^7,8^, which was originally identified in flagella of the green alga *Chlamydomonas* and is often referred to as ‘IFT particles’ or ‘IFT trains’^9–11^. Within the IFT machinery, the IFT-B complex, which is composed of 16 subunits, mediates anterograde trafficking driven by the kinesin-II motor, and the export of ciliary membrane proteins across the TZ together with the BBSome. On the other hand, the IFT-A complex, which is composed of six subunits, mediates retrograde trafficking driven by dynein-2 (also known as IFT dynein) and the import of ciliary GPCRs across the TZ together with the TULP3 adaptor^2,12,13^. In addition, recent studies in *Caenorhabditis elegans* suggested that the IFT-A complex and IFT dynein are required for the integrity and gating function of the TZ^14,15^.

Dynein-2/IFT dynein is a very large protein complex that is composed of five subunits specific to dynein-2 (the DYNC2H1 heavy chain, the WDR60 and WDR34 intermediate chains [recently renamed as DYNC2I1 and DYNC2I2, respectively], the DYNC2LI1 light intermediate chain [LIC], and the TCTEX1D2 light chain [recently renamed as DYNLT2B]), and three-types of light chains shared with the dynein-1 complex (DYNLL1/DYNLL2, DYNLRB1/DYNLRB2, and DYNLT1/DYNLT3)^12,16,17^. Biochemical and interactome analyses by us and others delineated the architectural model of the mammalian dynein-2 complex^18–21^, in which DYNC2LI1 forms a subcomplex with the N-terminal tail (nonmotor) region of DYNC2H1, which in turn interacts with WDR60 and WDR34. Our model is largely consistent with the recently clarified cryo-EM structure of the human dynein-2 complex^22^, in which two molecules of DYNC2H1 adopt asymmetric conformations in the tail region, with each DYNC2H1 molecule binding to DYNC2LI1, and either WDR60 or WDR34. Docking of the dynein-2 structure into the anterograde IFT train structure of *Chlamydomonas* flagella^23^ clarified by cryoelectron tomography suggested that each dynein-2 complex spans out multiple IFT-B repeats when it is transported as a cargo of the anterograde IFT train^17,22,24^. In agreement with the docking model, interactome analyses of WDR60 and WDR34 suggested that dynein-2 interacts with multiple IFT-B subunits^21^. Furthermore, it is interesting to note that while this study was in progress, the DYNC2LI1 ortholog in *Chlamydomonas* was reported to interact with the IFT-B subunit IFT54, and the DYNC2LI1–IFT54 interaction was suggested to be crucial for the transport of dynein-2 as a cargo of the anterograde IFT train^25^.

In line with the cooperative role of the IFT-A and dynein-2 complexes in retrograde trafficking, mutations of all IFT-A subunits and dynein-2-specific subunits are known to cause skeletal ciliopathies characterized by a narrow thorax and polydactyly, generally termed short-rib thoracic dystrophy (SRTD; OMIM 208,500), including short rib-polydactyly syndrome (SRPS), Jeune asphyxiating thoracic dystrophy (JATD), Ellis-van Creveld syndrome (EvC; OMIM 225,500), and cranioectodermal dysplasia (CED; OMIM 218,330)^6,26–31^. We recently demonstrated the molecular basis of the ciliary defects caused by CED-associated variations of the IFT-A subunits IFT122 and IFT144/WDR19^32,33^. In this study, we focused on *DYNC2LI1*, which is the causative gene of SRTD15 (OMIM 617,088), as several pathogenic biallelic variations were reported^34–37^. We here show that several DYNC2LI1 variants have reduced abilities to bind DYNC2H1 and WDR60. More importantly, we found that in *DYNC2LI1*-knockout (KO) cells, the expression of a single deletion variant in combination with a missense variant causes substantial ciliary defects, but not in combination with wild-type (WT) DYNC2LI1.

## Results

### Variations of DYNC2LI1 found in SRTD individuals affect its interactions with DYNC2H1 and WDR60

**Figure 1:**
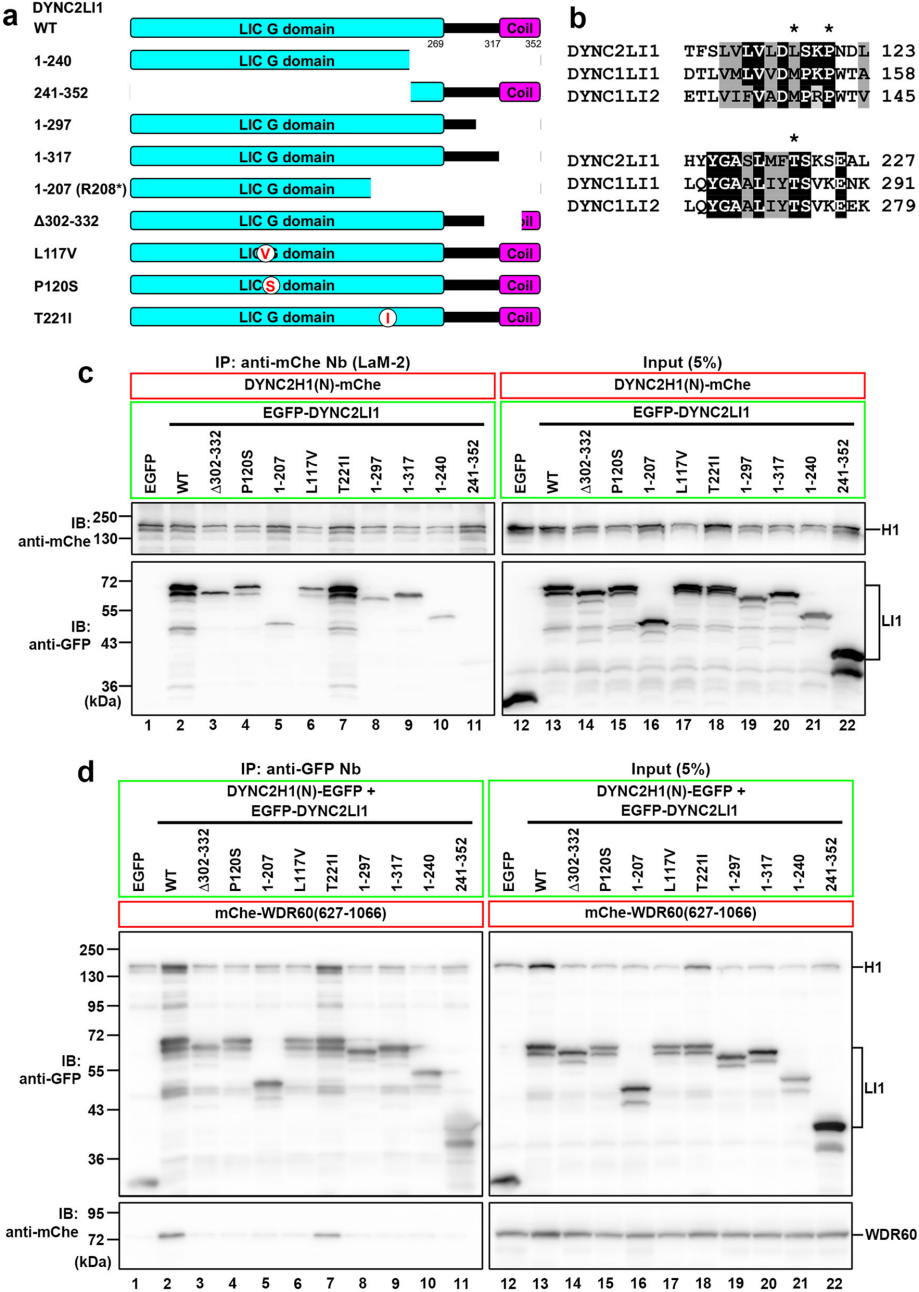
Multiple DYNC2LI1 variants found in SRTD individuals have compromised interactions with DYNC2H1 and WDR60. (**a**) Schematic representation of the DYNC2LI1 constructs used in the interaction experiments shown in (**c**,**d**) and in Fig. S1a,b. (**b**) Sequence alignment of the conserved regions among human DYNC2LI1, DYNC1LI1, and DYNC1LI2. Amino acid residues identical between DYNC2LI1 and DYNC1LI1 and/or DYNC1LI2 are shown in a black background, and those with conservative substitutions are shown in a grey background. The positions of L117, P120, and T221 are indicated by asterisks. (**c**) Lysates prepared from HEK293T cells coexpressing EGFP-fused DYNC2LI1 constructs, as indicated, and DYNC2H1(N)-mChe were subjected to immunoprecipitation using GST-tagged anti-mChe Nb (LaM-2 version), followed by immunoblotting analysis using anti-mChe and anti-GFP antibodies. (**d**) Lysates of cells coexpressing the DYNC2H1(N)-EGFP and EGFP-fused DYNC2LI1 constructs, as indicated, together with mChe-WDR60(627–1066), were subjected to immunoprecipitation using GST–anti-GFP Nb, followed by immunoblotting analysis using anti-mChe and anti-GFP antibodies.

As DYNC2LI1 interacts directly with the DYNC2H1 heavy chain^18,22^, we first determined which region(s) of the DYNC2LI1 protein participates in its interaction with DYNC2H1. The previously revealed X-ray crystallographic structure of the dynein-1 LIC of thermophilic yeast and biochemical experiments using human DYNC1LI1 demonstrated that DYNC1LI1 interacts with DYNC1H1 via the N-terminal Ras-like G domain (Fig. 1a), which is evolutionally conserved among the LICs of the dynein-1 and dynein-2 complexes (Fig. 1b)^38^. In addition, the cryo-EM structure of the dynein-2 complex indicated that both the G domain and the C-terminal coil region of DYNC2LI1 participate in its interaction with DYNC2H1^22^. In addition, there is a relatively long α-helical coil region (residues 318–352) at the C-terminus of DYNC2LI1 (Fig. 1a)^35^. We therefore made some DYNC1LI1 constructs with truncations from the C-terminus, and analyzed their interactions with the N-terminal tail region (residues 1–1090) of DYNC2H1 [hereafter referred to as DYNC2H1(N)]. Lysates prepared from HEK293T cells coexpressing DYNC2H1(N)-mCherry (mChe) and any of the DYNC2LI1 constructs fused to EGFP were subjected to immunoprecipitation with glutathione *S*-transferase (GST)-fused anti-mChe nanobodies (Nb) (LaM-2 version)^33^ prebound to glutathione-Sepharose beads, followed by SDS-PAGE and immunoblotting analysis using anti-mChe and anti-GFP antibodies. As shown in Fig. 1c, EGFP-DYNC2LI1(WT), but not EGFP alone, was coimmunoprecipitated with DYNC2H1(N)-mChe (compare lanes 1 and 2). The DYNC2LI1(1–317) construct, which lacks the C-terminal coil region (Fig. 1a), was much less efficiently coprecipitated with DYNC2H1(N)-mChe than DYNC2LI1(WT) (Fig. 1c, compare lanes 2 and 9). The amounts of the other truncation constructs, DYNC2LI1(1–297) and DYNC2LI1(1–240), coprecipitated with DYNC2H1(N)-mChe were also substantially lower than that of DYNC2LI1(WT) (compare lanes 8 and 10 with lane 2). On the other hand, the C-terminal construct DYNC2LI1(241–352) was not coprecipitated with DYNC2H1(N)-mChe (lane 11). These results are consistent with the dynein-2 cryo-EM structure, which indicates the contribution of the C-terminal coil region as well as the G domain of DYNC2LI1 to its interaction with DYNC2H1^22^.

To date, four studies have identified compound heterozygous variations in *DYNC2LI1* in individuals showing phenotypes of the skeletal ciliopathies (Table S1)^34–37^. One case study reported combinations of a missense variant p.(Leu117Val) [hereafter referred to as DYNC2LI1(L117V) for the variant DYNC2LI1 protein] with the deletion/truncation variant, p.(Ser302_Ile332del) [hereafter DYNC2LI1(Δ302–332)] or p.(Trp124*)^36^ in individuals showing phenotypes of the skeletal ciliopathies. In two other case studies, affected individuals were found to have combinations of the missense variant, p.(Thr221Ile) [hereafter DYNC2LI1(T221I)], and the truncation variant, p.(Arg208*) [hereafter DYNC2LI1(1–207)], p.(Val141*), or p.(Met1?), which has a mutation at the initiation codon^35,37^. While this study was underway, a study reported a combination of the missense variant, p.(Pro120Ser) [hereafter DYNC2LI1(P120S)] and a truncation variant, p.(Lys310*) in an affected individual^34^. Among the deletion/truncation variants, we selected DYNC2LI1(Δ302–332) and DYNC2LI1(1–207), and analyzed their interactions with DYNC2H1. We were also interested in three missense variants, DYNC2LI1(L117V), DYNC2LI1(P120S), and DYNC2LI1(T221I), as these mutated residues are conserved not only in DYNC2LI1 but also in the dynein-1 LICs, DYNC1LI1 and DYNC1LI2 (Fig. 1b).

As expected from the analysis of the C-terminal truncation variants described above, DYNC2LI1(Δ302–332) and DYNC2LI1(1–207) were found to have substantially reduced abilities to interact with DYNC2H1, compared with DYNC2LI1(WT) (Fig. 1c, compare lanes 3 and 5 with lane 2). Among the missense variants, DYNC2LI1(T221I) retained DYNC2H1-binding ability to a level comparable to that of DYNC2LI1(WT) (lane 7), whereas DYNC2LI1(L117V) and DYNC2LI1(P120S) had reduced DYNC2H1-binding ability (lanes 4 and 6). It is of note that the amount of the DYNC2H1(N)-mChe protein tends to be reduced when coexpressed with any of the DYNC2LI1 constructs with reduced interacting abilities (Fig. 1c, input panel); therefore, DYNC2H1(N) might be unstable in the absence of its efficient interaction with DYNC2LI1 (also see below).

As our previous study indicated that a subcomplex of DYNC2H1 and DYNC2LI1 efficiently interacts with the C-terminal WD40 repeat region of WDR60/DYNC2I1^18^, we then analyzed the interactions of WDR60(627–1,066) with a combination of DYNC2H1(N) and any of the DYNC2LI1 constructs. The results shown in Fig. 1d correlated well with those for the binary interactions between DYNC2H1(N) and the DYNC2LI1 construct shown in Fig. 1c; namely, mChe-WDR60(627–1,066) was coimmunoprecipitated with DYNC2H1(N)-EGFP when combined with EGFP-DYNC2LI1(T221I) as efficiently as when combined with EGFP-DYNC2LI1(WT) (Fig. 1d, lanes 2 and 7); in striking contrast, coprecipitation of mChe-WDR60(627–1,066) was abolished when DYNC2H1(N)-EGFP was combined with any other DYNC2LI1 construct (lanes 3–6 and lanes 8–11). Thus, it is likely that WDR60 interacts efficiently with the DYNC2H1–DYNC2LI1 dimer but not with DYNC2H1 alone. Again, it is of note that DYNC2H1(N) appeared to be unstable in the absence of its efficient interaction with DYNC2LI1 (Fig. 1d, input panel), although we did not pursue this issue further.

Our previous studies also suggested that although DYNC2H1 alone can interact with the WD40 repeat region of WDR34, its efficient interaction with WDR34 requires its subcomplex formation with DYNC2LI1^18,19^. We therefore analyzed the interactions of WDR34(106–536) with a combination of DYNC2H1(N) and any of the DYNC2LI1 constructs. However, in contrast to the results obtained for WDR60 (Fig. 1d), the amount of mChe-WDR34(106–536) coimmunoprecipitated with DYNC2H1(N)-EGFP did not substantially vary in the presence of any of the coexpressed EGFP-DYNC2LI1 constructs (Fig. S1a). Thus, it is likely that WDR34 interacts primarily with DYNC2H1, and that the DYNC2LI1 variations found in SRTD do not considerably affect the interaction of WDR34 with DYNC2H1–DYNC2LI1.

While this study was in progress, a study using *Chlamydomonas* reported that IFT54, which is a subunit of the IFT-B complex, binds to IFT dynein via the DYNC2LI1 ortholog, and suggested that the IFT54–DYNC2LI1 interaction is crucial for anterograde trafficking of the dynein-2 complex, as a cargo of the IFT machinery^25^. We therefore analyzed the interactions of human IFT54 with the various DYNC2LI1 constructs. As shown in Fig. S1b, we confirmed that mChe-IFT54 coimmunoprecipitated EGFP-DYNC2LI(WT) (lane 2). Regarding the DYNC2LI1 variants, DYNC2LI1(241–352) (lane 11) lacked the ability to interact with IFT54, and DYNC2LI1(1–207) and DYNC2(1–240) (lanes 5 and 10) appeared to have substantially reduced IFT54-binding ability. These results indicate that DYNC2LI1 interacts with IFT54 mainly via the G domain, and that the missense variations of DYNC2LI1 found in SRTD do not substantially affect its interaction with IFT54.

### Defects of ciliary protein trafficking in DYNC2LI1-KO cells exogenously expressing DYNC2LI1 variants

**Figure 2:**
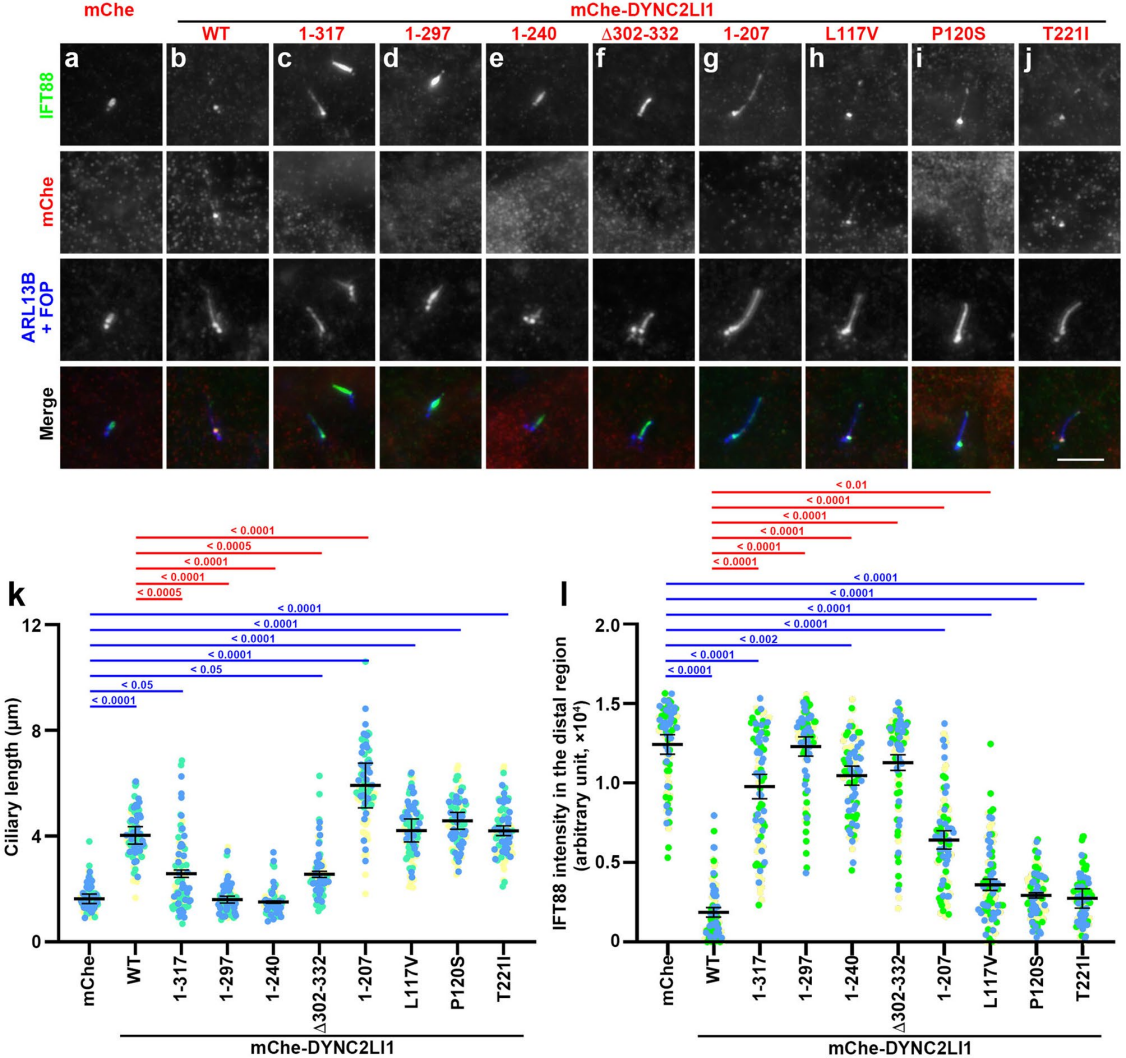
Effects of the expression of DYNC2LI1 variants on ciliogenesis and IFT88 localization in *DYNC2LI1*-KO cells. (**a**–**j**) *DYNC2LI1*-KO cells expressing mChe (**a**), or mChe-fused DYNC2LI1 constructs as indicated (**b**–**j**) were serum-starved for 24 h to induce ciliogenesis, and immunostained with antibodies against IFT88 and ARL13B + FOP. Scale bar, 5 µm. (**k**) Ciliary lengths of individual cells were measured and expressed as scatter plots. (**l**) Relative staining intensities of IFT88 in the distal region of cilia were estimated and expressed as scatter plots. Different colored dots represent three independent experiments (*n* = 30 × 3). Horizontal lines are means, and error bars are SD. Statistical significances were calculated using one-way ANOVA followed by the Dunnett’s multiple comparison test.

To analyze the functional defects of the DYNC2LI1 variants, we first established *DYNC2LI1*-KO cells from human telomerase reverse transcriptase-immortalized retinal pigment epithelial 1 (hTERT-RPE1) cells using the CRISPR/Cas9 system^39^. The *DYNC2LI1*-KO cell line #DYNC2LI1-3–2 (Fig. S2a,b) was found to have very short cilia, when stained with antibodies against ARL13B (a marker of the ciliary membrane), acetylated α-tubulin (Ac-tubulin; a marker of the ciliary axoneme), and γ-tubulin (a marker of the basal body) (Fig. S2c,d; also see Fig. 2a). The ciliogenesis defect observed in the #DYNC2LI1-3–2 cell line did not result from off-target effects of the CRISPR/Cas9 system, as the exogenous expression of mChe-DYNC2LI1(WT), but not mChe alone, restored normal ciliary length (compare Fig. 2a,b; also see Fig. 2k). The very short cilia-phenotype of *DYNC2LI1*-KO cells was essentially the same as those of *C. elegans* and *Chlamydomonas* depleted of DYNC2LI1 orthologs^40,41^; loss of the dynein-2 LIC destabilizes the heavy chain and blocks retrograde IFT, resulting in impaired recycling of IFT components and defect in axoneme extension, like loss of the heavy chain itself^42^.

As for the C-terminal truncation variants, the exogenous expression of mChe-DYNC2LI1(1–317) in *DYNC2LI1*-KO cells partially restored ciliogenesis (Fig. 2c), whereas the expression of mChe-fused DYNC2LI1(1–297) or DYNC2LI1(1–240) did not (Fig. 2d,e,k). In addition, IFT88 appeared to be enriched within very short cilia (see below).

We then analyzed whether the DYNC2LI1 variants found in SRTD individuals are able to rescue the ciliogenesis defect of *DYNC2LI1*-KO cells. The expression of mChe-DYNC2LI1(Δ302–332) partially rescued the ciliogenesis defect (Fig. 2f), as for the expression of mChe-DYNC2LI1(1–317) (Fig. 2c,k). Somewhat unexpected was the phenotype of *DYNC2LI1*-KO cells expressing mChe-DYNC2LI1(1–207) (Fig. 2g); these cells had cilia that were significantly longer than those expressing DYNC2LI1(WT) (Fig. 2k) (see "Discussion" Section). When missense SRTD variants, namely, DYNC2LI1(L117V), DYNC2LI1(P120S), and DYNC2LI1(T221I), were expressed in *DYNC2LI1*-KO cells, all the variants were found to generate cilia of normal length (Fig. 2h–j,k).

The localization of IFT88 (an IFT-B subunit) in *DYNC2LI1*-KO cells expressing DYNC2LI1 variants was also analyzed, as our previous studies showed that KO cells of other dynein-2 subunits demonstrated significant accumulation of IFT machinery components within cilia. This was also the case for *DYNC2LI1*-KO cells; *DYNC2LI1*-KO cells expressing mChe alone demonstrated the enrichment of IFT88 within short cilia, particularly in the distal region, whereas this enrichment was eliminated by the expression of mChe-DYNC2LI1(WT) (compare Fig. 2a,b; also see Fig. 2l). Ciliary IFT88 enrichment was not eliminated by the expression of DYNC2LI C-terminal truncation variants, i.e., DYNC2LI1(1–317), DYNC2LI1(1–297), or DYNC2LI1(1–240), or by the expression of DYNC2LI1(Δ302–332) (Fig. 2c–f,l). By contrast, the missense variants restored the normal IFT88 localization at the ciliary base (Fig. 2h–j), comparable to DYNC2LI1(WT) (Fig. 2l). In *DYNC2LI1*-KO cells expressing DYNC2LI1(1–207), the enrichment of IFT88 in cilia was partially reduced (Fig. 2g,l).

**Figure 3:**
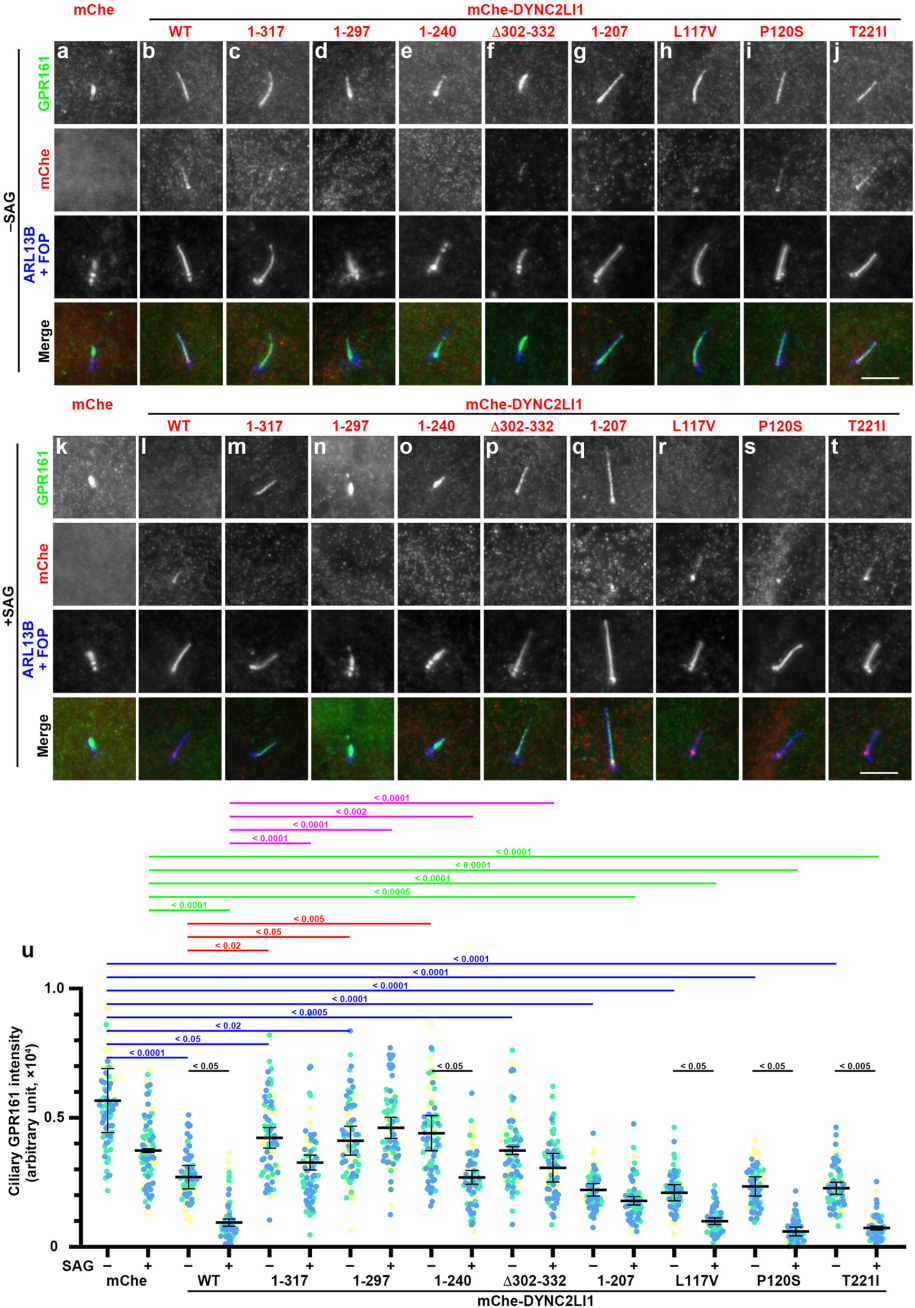
Effects of the expression of DYNC2LI1 variants on basal ciliary level and induced ciliary exit of GPR161 in *DYNC2LI1*-KO cells. (**a**–**t**) *DYNC2LI1*-KO cells expressing mChe (**a**, **k**), or mChe-fused DYNC2LI1 constructs as indicated (**b**–**j**, **l**–**t**) were serum-starved for 24 h to induce ciliogenesis, cultured in the absence (**a**–**j**; –SAG) or presence (**k**–**t**; + SAG) of SAG for a further 24 h, and immunostained with antibodies against GPR161 and ARL13B + FOP. Scale bars, 5 µm. (**u**) Relative ciliary staining intensities of GPR161 were estimated and expressed as scatter plots. Different colored dots represent three independent experiments (*n* = 30 × 3), horizontal lines are means, and error bars are SD. Statistical significances among multiple cell lines were calculated using one-way ANOVA followed by the Dunnett’s multiple comparison test, and those between two groups (–SAG and + SAG) were calculated using the Student *t*-test.

We then analyzed the localization of GPR161 in *DYNC2LI1*-KO cells expressing the DYNC2LI1 variants; GPR161 is localized on the ciliary membrane under basal conditions to suppress Hh signaling, and it exits cilia upon Hh pathway stimulation^3^. In *DYNC2LI1*-KO cells expressing mChe-DYNC2LI1(WT), GPR161 was found within cilia under basal conditions and became undetectable when the cells were treated with Smoothened agonist (SAG) (Fig. 3b,l). By contrast, in *DYNC2LI1*-KO cells expressing mChe alone, GPR161 was retained within short cilia even in the presence of SAG (Fig. 3a, k), indicating that exit of GPR161 from cilia upon Hh signaling activation is suppressed in *DYNC2LI1*-KO cells. It is also notable that the relative staining intensity of ciliary GPR161 in mChe-expressing *DYNC2LI1*-KO cells was significantly higher than that in mChe-DYNC2LI1(WT)-expressing *DYNC2LI1*-KO cells under basal conditions (Fig. 3a,b; also see Fig. 3u), suggesting that constitutive GPR161 exit is also suppressed in the absence of DYNC2LI1. Essentially the same results were obtained for *DYNC2LI1*-KO cells expressing DYNC2LI1(1–317), DYNC2LI1(1–297), DYNC2LI1(1–240), or DYNC2LI1(Δ302–332); namely, GPR161 was significantly retained within short cilia even when the cells were treated with SAG (Fig. 3c–f,m–p,u). In *DYNC2LI1*-KO cells expressing DYNC2LI1(1–207), the basal ciliary level of GRP161 was relatively low, but the level was not significantly decreased even upon SAG treatment (Fig. 3g,q,u). By contrast, in *DYNC2LI1*-KO cells expressing any of the missense variants, the ciliary GRP161 level was significantly decreased when the cells were treated with SAG (Fig. 3h–j,r–t,u), as in DYNC2LI1(WT)-expressing cells (Fig. 3b, l).

### Specific combinations of DYNC2LI1 variants are unable to rescue the ciliary defects in DYNC2LI1-KO cells

As described above, all the reported individuals with skeletal ciliopathies caused by variations in *DYNC2LI1* have compound heterozygous mutations (Table S1). Therefore, we then addressed how combinations of the DYNC2LI1 variants contribute to the ciliary defects. To this end, we expressed the DYNC2LI1 variants in *DYNC2LI1*-KO cells in the combinations found in compound heterozygous individuals, and analyzed the phenotypes of these cells.

**Figure 4:**
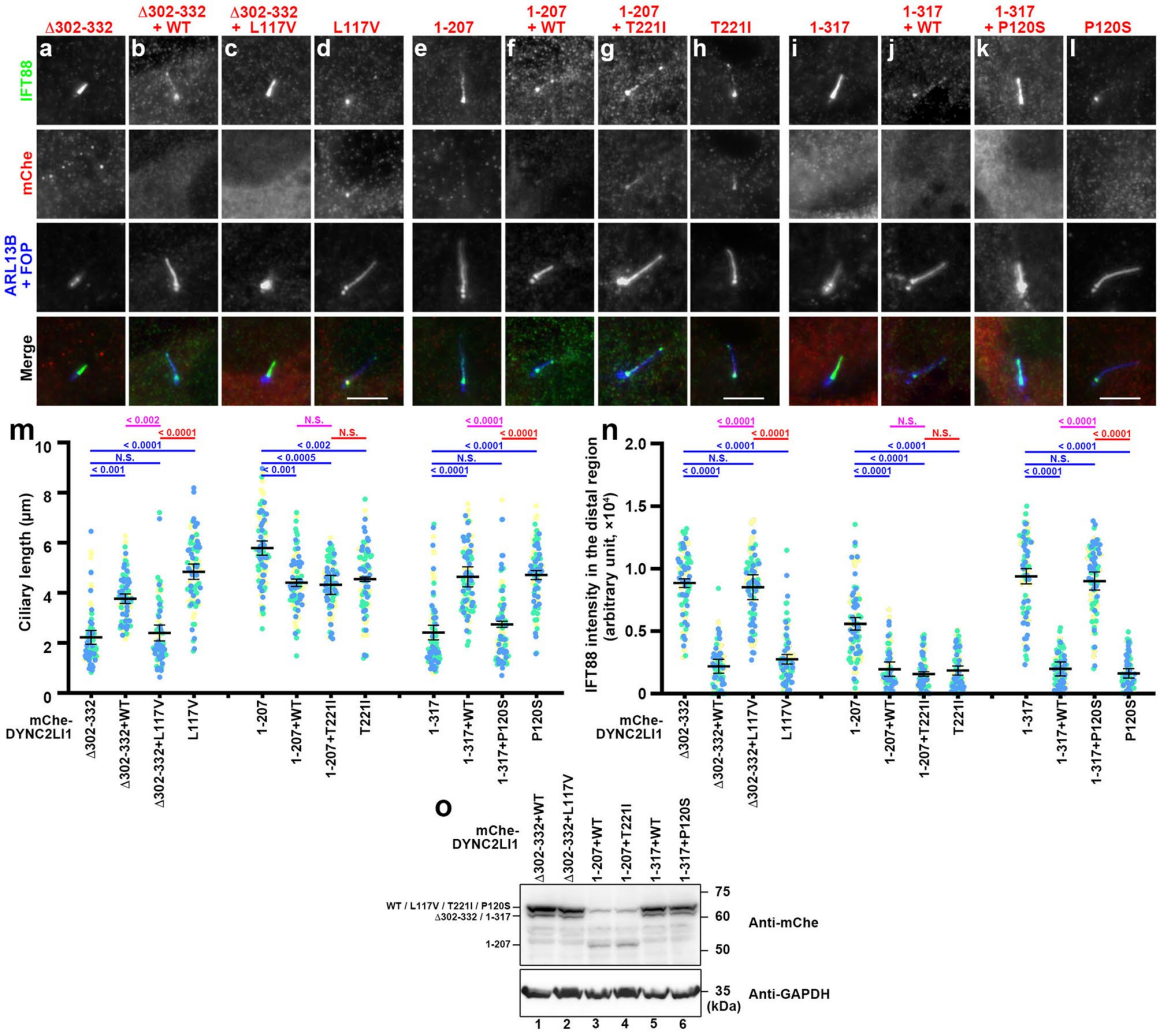
Specific combinations of DYNC2LI1 variants are unable to rescue defects in ciliogenesis and IFT88 localization in *DYNC2LI1*-KO cells. (**a**–**l**) *DYNC2LI1*-KO cells expressing the indicated mChe-fused DYNC2LI1 construct or the indicated combinations of mChe-fused DYNC2LI1 constructs were serum-starved for 24 h and immunostained with antibodies against IFT88 and ARL13B + FOP. Scale bars, 5 µm. (**m**,**n**) Ciliary lengths of individual cells and staining intensities for IFT88 in the distal region of cilia in the experiments shown in (**a**–**l**) were measured and expressed as scatter plots, and analyzed as described in the legend to Fig. 2k,l, respectively. (**o**) Immunoblotting analysis of equivalent amounts of cell lysates coexpressing the indicated combinations of mChe-fused DYNC2LI1 constructs, with an anti-mChe antibody.

We first analyzed the effects of expression of the combination of DYNC2LI1(L117V) and DYNC2LI1(Δ302–332) in *DYNC2LI1*-KO cells, which mimics the cellular situation of an SRPS individual reported by Taylor et al*.*^36^. It is noteworthy that the same study reported the p.(Leu117Val) variant [in combination with p.(Trp124*)] in one other SRPS individual and the p.(Ser302_Ile332del) variant [in combination with p.(Glu335*)] in another individual^36^. As described above (Fig. 2), *DYNC2LI1*-KO cells expressing mChe-DYNC2LI1(Δ302–332) showed defects in ciliogenesis and ciliary IFT88 level (Fig. 4a), whereas those expressing mChe-DYNC2LI1(L117V) did not show either defect (Fig. 4d). When mChe-fused DYNC2LI1(Δ302–332) and DYNC2LI1(WT) were coexpressed in *DYNC2LI1*-KO cells, a situation that mimics cells of a healthy parent of an affected individual, both normal ciliary length and low ciliary IFT88 level were significantly restored (Fig. 4b,m,n). In striking contrast, when DYNC2LI1(Δ302–332) was coexpressed together with DYNC2LI1(L117V), ciliogenesis was not significantly recovered and ciliary IFT88 enrichment was not significantly rescued (Fig. 4c,m,n). Note that we confirmed the expression of both mChe-DYNC2LI1(Δ302–332) and mChe-DYNC2LI1(L117V) by immunoblotting analysis of the cell lysates (Fig. 4o, lane 2).

**Figure 5:**
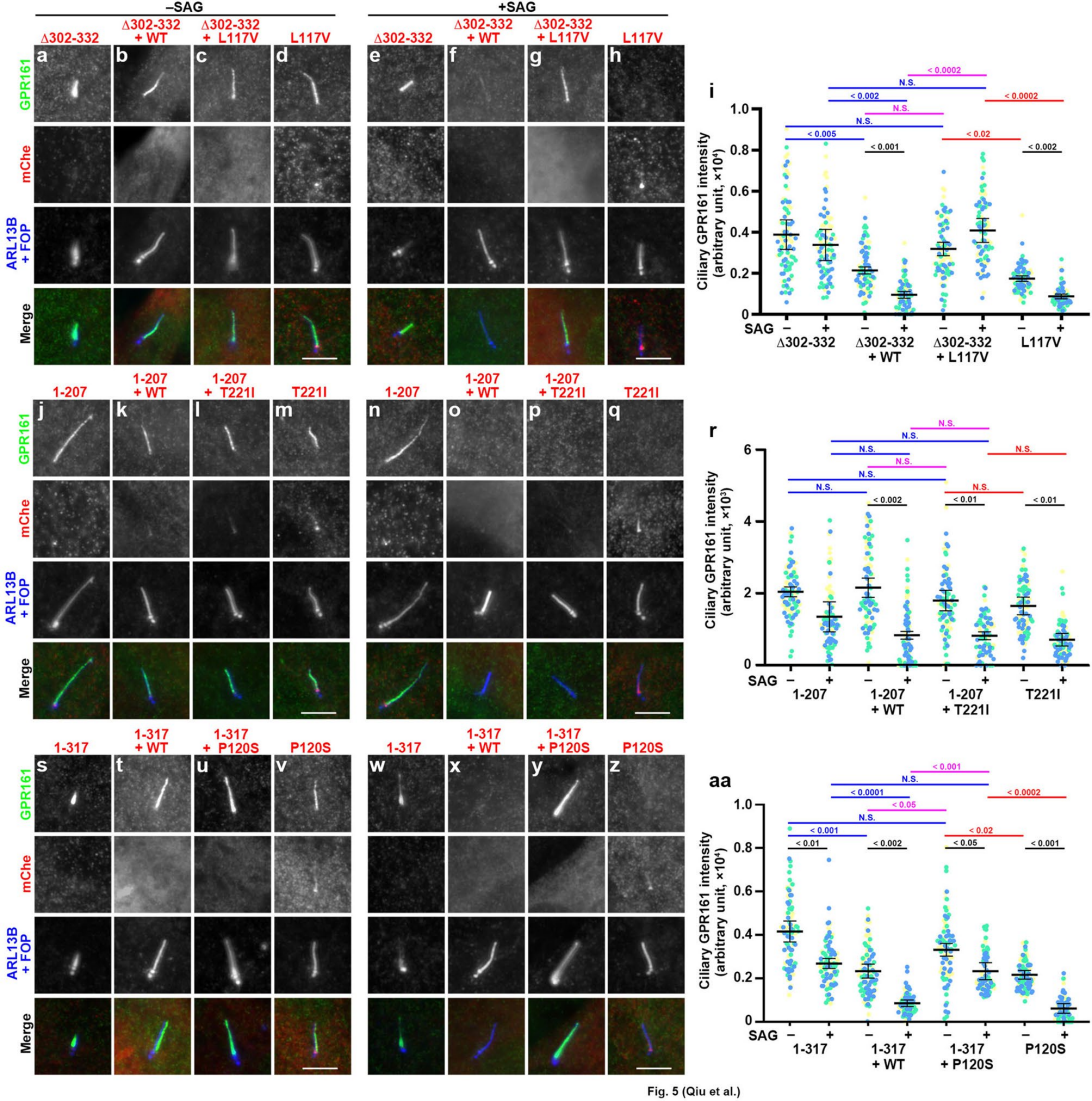
Specific combinations of DYNC2LI1 variants are unable to rescue defects in the induced exit of GPR161 from cilia in *DYNC2LI1*-KO cells. (**a**–**h**, **j**–**q**, **u**–**z**) *DYNC2LI1*-KO cells expressing the indicated mChe-fused DYNC2LI1 construct or the indicated combinations of mChe-fused DYNC2LI1 constructs were serum-starved for 24 h to induce ciliogenesis, cultured in the absence (**a**–**d**, **j**–**m**, **s**–**v**; –SAG) or presence (**e**–**h**, **n**–**q**, **w**–**z**; + SAG) of SAG for a further 24 h, and immunostained with antibodies against GPR161 and ARL13B + FOP. Scale bars, 5 µm. (i, r, aa) Relative ciliary staining intensities of GPR161 in the experiments shown in a–h, j–q, and s–z, respectively, were estimated and expressed as scatter plots, and analyzed as described in the legend to Fig. 3u.

We also compared the ciliary localizations of GPR161 in the presence and absence of SAG treatment in *DYNC2LI1*-KO cells expressing a combination of DYNC2LI1(Δ302–332) and either DYNC2LI1(WT) or DYNC2LI1(L117V). Again, when expressed alone in *DYNC2LI1*-KO cells, DYNC2LI1(L117V) (Fig. 5d,h), but not DYNC2LI1(Δ302–332) (Fig. 5a, e, i), restored the ciliary exit of GPR161 in response to SAG treatment. Combinatorial expression of DYNC2LI1(Δ302–332) and DYNC2LI1(WT) also restored the exit of GRP161 from cilia upon SAG treatment (Fig. 5b,f,i). In striking contrast, in *DYNC2LI1*-KO cells coexpressing DYNC2LI1(Δ302–332) together with DYNC2LI1(L117V), GPR161 was significantly retained within cilia even when the cells were stimulated with SAG (Fig. 5c,g,i). Thus, ciliary length, ciliary localization of IFT88, and the induced exit of GPR161 were abnormal in *DYNC2LI1*-KO cells coexpressing DYNC2LI1(Δ302–332) together with the missense variant DYNC2LI1(L117V), but not together with DYNC2LI1(WT). These observations are consistent with a previous study showing that in fibroblasts derived from an SRPS individual with heterozygous alleles of DYNC2LI1(Δ302–332) and DYNC2LI1(L117V), IFT components including IFT88 were accumulated within cilia^36^.

Another case study reported an individual with skeletal ciliopathy with a spectrum between EvC and JATD caused by compound heterozygous variants, p.(Thr221Ieu) and p.(Arg208*) (Table S1)^35^. A subsequent study reported EvC patients with combinations of p.(Thr221Ieu) and either p.(Val141*) or an initiation codon mutant of DYNC2LI1 (Table S1)^37^. Thus, DYNC2LI1(T221I) is likely to be crucial for the pathogenesis of EvC. We therefore analyzed the effects of the expression of DYNC2LI1(T221I) in combination with the deletion variant DYNC2LI1(1–207) in *DYNC2LI1*-KO cells. As described above (Fig. 2), *DYNC2LI1*-KO cells expressing mChe-DYNC2LI1(1–207) alone demonstrated relatively long cilia with substantial accumulation of IFT88 within cilia (Fig. 4e), and impaired exit of GRP161 in response to SAG treatment (Fig. 5j,n). On the other hand, the phenotype of *DYNC2LI1*-KO cells expressing mChe-DYNC2LI1(T221I) appeared to be normal (Fig. 4h, and Fig. 5m, q; also see Figs. 4m,n, and 5r). When DYNC2LI1(1–207) was coexpressed with either DYNC2LI1(WT) or DYNC2LI1(T221I) in *DYNC2LI1*-KO cells, their phenotypes appeared to be normal (Figs. 4f, g; and 5k, l,o,p; also see Figs. 4m,n and 5r) and were indistinguishable from those expressing DYNC2LI1(WT) alone (Figs. 2 and 3). Thus, the pathogenic combination of EvC [DYNC2LI1(1–207) and DYNC2LI1(T221I)] did not apparently affect ciliogenesis or ciliary protein localization (see "Discussion" Section).

While this study was in progress, a case study reported a combination of the DYNC2LI1 variants, p.(Pro120Ser) and p.(Lys310*), in a fetus with SRPS-like phenotypes (Table S1)^34^. We therefore analyzed the effects of this combination in *DYNC2LI1*-KO cells. However, in this experiment, we used DYNC2LI1(1–317) instead of DYNC2LI1(K310*), as we thought that cells expressing DYNC2LI1(1–317) would most closely reflect the situation of those expressing DYNC2LI1(K310*). As described above (Fig. 2), *DYNC2LI1*-KO cells expressing DYNC2LI1(1–317) alone demonstrated relatively short cilia, significant enrichment of IFT88 within cilia (Fig. 4i), and impaired exit of GPR161 in response to SAG treatment (Fig. 5s,w), whereas those expressing DYNC2LI1(P120S) were normal with respect to cilia length, IFT88 localization (Fig. 4l), and GPR161 exit (Fig. 5v,z). *DYNC2LI1*-KO cells coexpressing DYNC2LI1(1–317) and DYNC2LI1(WT) also appeared normal regarding cilia length and IFT88 localization (Fig. 4j,m,n) and the SAG-induced exit of GPR161 (Fig. 5t,x,aa). However, *DYNC2LI1*-KO cells coexpressing DYNC2LI1(1–317) and DYNC2LI1(P120S) had short cilia and considerable enrichment of IFT88 within cilia (Fig. 4k,m,n) and significant impairment of GRP161 exit upon SAG treatment (Fig. 5u,y,aa). Thus, *DYNC2LI1*-KO cells coexpressing DYNC2LI1(1–317) together with DYNC2LI1(L117V), but not with DYNC2LI1(WT), were abnormal with regard to their cilia length, ciliary IFT88 localization, and GPR161 exit from cilia.

## Discussion

We here demonstrated the molecular and cellular basis of the ciliary defects in SRPS skeletal ciliopathy caused by compound heterozygous variations of the dynein-2 LIC, DYNC2LI1. Namely, we showed that combinatorial expression of a DYNC2LI1 variant with an extensive deletion [DYNC2LI1(Δ302–332) or DYNC2LI1(1–317)] together with a missense variant [DYNC2LI1(L117V) or DYNC2LI1(P120S)], but not with DYNC2LI1(WT), in *DYNC2LI1*-KO cells causes defects in cilia biogenesis, retrograde trafficking of the IFT machinery, and exit of GPR161 from cilia upon stimulation of the Hh signaling pathway (Figs. 4 and 5). These observations are consistent with ciliary defects observed in fibroblasts derived from SRPS individuals^36^, and are in line with the fact that SRPS individuals with compound heterozygous variations demonstrate severe symptoms, whereas their parents with one of the variations are healthy^34,36^. On the other hand, expression of either of the missense variants DYNC2LI1(L117V) or DYNC2LI1(P120S) in *DYNC2LI1*-KO cells was able to rescue the ciliary defects, as with DYNC2LI1(WT) (Figs. 4 and 5). However, we found that these missense variants are indeed compromised with regard to their interactions with DYNC2H1 and WDR60 (Fig. 1c, d). Thus, these missense variations might have subtle effects on the overall function of dynein-2, and lead to an abnormal ciliary phenotype in a context-dependent manner.

In contrast to the compound heterozygous variations found in SRPS individuals, the combination of an extensive deletion and a missense variant [DYNC2LI1(1–207)/DYNC2LI1(T221I)] found in EvC individuals did not lead to apparent ciliary defects when expressed in *DYNC2LI1*-KO cells. EvC appears to be a milder subtype of SRTD than SRPS; in contrast to the prenatal lethality of SRPS individuals, EvC individuals are often able to survive to adulthood. Unexpectedly, DYNC2LI1(1–207), albeit the extensive deletion, caused cilia elongation, rather than shortening, in *DYNC2LI1*-KO cells. Although we do not know the exact reason for the cilia elongation in DYNC2LI1(1–207)-expressing cells, this may be due to a subtle difference in the interaction(s) with other dynein-2 subunits between DYNC2LI1(1–207) and other deletion constructs. On the other hand, even though the DYNC2LI1(T221I) variants was reported in three compound heterozygous EvC patients^37^, we did not detect any apparent defects in the interactions of DYNC2LI1(T221I) with DYNC2H1 and WDR60, in contrast to the other missense variants analyzed, i.e., DYNC2LI1(L117V) and DYNC2LI1(P120S) (Fig. 1). Thus, unlike in the case of SRPS, just a subtle defect in the interaction(s) of DYNC2LI1(T221I) with some other protein(s) might affect the trafficking and/or localization of ciliary proteins, which we did not analyze, and may be responsible for the abnormalities observed in EvC individuals. For example, to date, we have not been able to detect an interaction between the dynein-2 complex and the IFT-A complex, even though involvement of the IFT-A complex in retrograde trafficking driven by the dynein-2 motor entails an interaction(s) between them. Such an interaction might take place after the dynein-2 complex is transported to the ciliary tip as an anterograde IFT cargo (see below).

In addition to DYNC2LI1, variations of other dynein-2 subunits and subunits of the IFT-A complex are known to cause SRTD^28^. As the IFT-A complex together with the dynein-2 complex mediates retrograde ciliary protein trafficking, impaired retrograde trafficking is implicated in the etiology of the skeletal ciliopathies. However, these are phenotypically diverse, and even in the same proteins, different variations and different combinations of variations can cause different subsets of ciliopathies. For example, we have recently shown that a combination of the missense variant of IFT144, p.(Leu710Ser) and the C-terminally truncated variant, p. (Arg1103*), a combination which is found in CED individuals^43^, exacerbated ciliogenesis defects when expressed in *IFT144*-KO cells, whereas expression of the missense variant alone in *IFT144*-KO cells, which mimics the cellular situation of recessive retinitis pigmentosa^44^, rescued the ciliary defects, as with the expression of IFT144(WT)^33^. Thus, the severity of the autosomal recessive ciliopathies appears to be associated with context-dependent mechanisms, in which one variant can lead to severe ciliary defects in combination with a hypomorphic variant.

To achieve its function as a retrograde motor for the IFT machinery, the dynein-2 complex must be transported to the ciliary tip as an anterograde IFT cargo. The cryo-EM structure of the human dynein-2 complex, in conjunction with the cryoelectron tomographic structure of *Chlamydomonas* anterograde IFT trains, suggested that the dynein-2 complex and the IFT-B complex interact with each other via multiple sites^17,22–24^. In addition, a study using *Chlamydomonas* demonstrated that the IFT-B subunit IFT54 directly binds to IFT dynein via its LIC subunit^25^. We here confirmed the DYNC2LI1–IFT54 interaction and that some DYNC2LI1 variants had reduced ability to interact with IFT54 (Fig. S1b). In view of the extensive contacts, however, there may be additional interactions between dynein-2 and the IFT-B subunits, and some ciliopathy variations of these subunits impair the dynein-2–IFT-B interactions. Furthermore, dynein-2 must be transported as an anterograde cargo in an autoinhibited state to avoid a tug-of-war between kinesin and dynein^17,22,45^, and the above *Chlamydomonas* study suggested that IFT54 interacts not only with IFT dynein but also with heterotrimeric kinesin-II. As it is possible that the short cilia phenotype may have resulted from an increased tug-of-war between kinesin and dynein, the difference between the expression of DYNC2LI1(1–317)/DYNC2LI1(1–297)/DYNC2LI1(1–240) and that of DYNC2LI1(1–207), which results in short and long cilia, respectively (Fig. 2), may be owing to the differential abilities of these DYNC2LI1 constructs to interfere with the autoinhibition of dynein-2. Therefore, an interesting issue to be addressed in the future is whether variations in the dynein-2 subunits affect the autoinhibited state.

## Methods

### Plasmids, antibodies, and reagents

Expression vectors for DYNC2LI1 and other dynein-2 subunits used in this study are listed in Table S2; some of them were constructed in our previous studies^18,19^. The antibodies used in this study are listed in Table S3. GST-tagged anti-GFP Nb and anti-mChe Nb (LaM-2 version) prebound to glutathione–Sepharose 4B beads (GE Healthcare) were prepared as described previously^33,46,47^. SAG and polyethylenimine Max were purchased from Enzo Life Sciences and Polysciences, respectively.

### Coimmunoprecipitation analyses

Coimmunoprecipitation analyses were performed based on the procedures previously described for the visible immunoprecipitation assay^46,48,49^. In brief, approximately 1.2 × 10^6^ HEK293T cells (RBC2202; RIKEN BioResource Research Center) were plated onto six-well plates. The next day, cells were transfected with EGFP and mChe fusion constructs using polyethylenimine Max (20 µg) and cultured in DMEM with high glucose supplemented with 5% fetal bovine serum (FBS) for 24 h. The cells were then suspended in 250 µL of HMDEKN cell lysis buffer (10 mM HEPES [pH 7.4], 5 mM MgSO_4_, 1 mM DTT, 0.5 mM EDTA, 25 mM KCl, and 0.05% NP-40) containing EDTA-free protease inhibitor cocktail (Nacalai Tesque), placed on ice for 20 min, and centrifuged at 16,100 × *g* for 15 min at 4 °C in a microcentrifuge. The supernatants (200 µL) were then incubated with 5 µL of GST-tagged anti-mChe Nb (LaM-2) or anti-GFP Nb prebound to glutathione–Sepharose 4B beads at 4 °C for 1 h, or for 3 h in the IFT54 experiments. The beads were washed three times with 180 µL of lysis buffer, boiled in SDS-PAGE sample buffer, and the proteins were separated by SDS-PAGE and electroblotted onto an Immobilon-P membrane (Merck Millipore). The membrane was then blocked in 5% skimmed milk and incubated sequentially with primary antibody and peroxidase-conjugated secondary antibody. Protein bands were detected using a Chemi-Lumi One L kit (Nacalai Tesque).

### Establishment of DYNC2LI1-KO cells

The strategy for the disruption of genes in hTERT-RPE1 cells (CRL-4000, American Type Culture Collection) by the CRISPR/Cas9 system using homology-independent DNA repair (version 2 method) was performed as previously described^39^, with minor modifications^50,51^. Briefly, single-guide RNA (sgRNA) sequences targeting the human *DYNC2LI1* gene (see Table S4) were designed using CRISPOR^52^. Double-stranded oligonucleotides for the target sequences were inserted into the all-in-one sgRNA expression vector peSpCAS9(1.1)-2 × sgRNA (Addgene 80768). Approximately 1.5 × 10^5^ hTERT-RPE1 cells were plated onto a 12-well plate. The next day, cells were transfected with the all-in-one vector and the donor knockin vector pDonor-tBFP-NLS-Neo(universal) (Addgene 80767) using X-tremeGENE9 Reagent (Roche Applied Science). After selection of the transfected cells in medium containing G418 (600 µg/mL), cells were isolated using an SH800 series cell sorter (SONY) at the Medical Research Support Center, Kyoto University. Genomic DNA extracted from the isolated cells were analyzed by PCR using GoTaq Master Mixes (Promega) and three sets of primers (Table S4) to distinguish the following three states of integration of the donor knockin vector: forward integration, reverse integration, and no integration with a small indel. The disruption of both *DYNC2LI1* alleles was confirmed by direct sequencing of the PCR products.

### Preparation of DYNC2LI1-KO cells expressing mChe-fused DYNC2LI1 constructs

The preparation of lentiviral vectors was performed as described previously^39,53^. Briefly, the pRRLsinPPT-based vectors for various DYNC2LI1 constructs were transfected into HEK293T cells together with the packaging plasmids [pRSV-REV, pMD2.g, and pMDLg/pRRE; kind gifts from Peter McPherson, McGill University^54^]. The culture medium was replaced 8 h after transfection, and collected between 24 to 48 h after transfection. The medium containing viral particles was passed through a 0.45-µm filter, and centrifuged at 32,000 × *g* at 4 °C for 4 h. Precipitated lentiviral particles were resuspended in DMEM/F-12 and stored at −80 °C until use. *DYNC2LI1*-KO cells expressing the mChe-fused DYNC2LI1 constructs were prepared by adding the lentiviral suspension to the culture medium followed by a 24-h incubation, and then subsequently used for analyses.

### Immunofluorescence analysis

Parental hTERT-RPE1 cells and *DYNC2LI1*-KO cells were cultured in DMEM/F-12 supplemented with 10% FBS and 0.348% sodium bicarbonate. To induce ciliogenesis, cells were grown to 100% confluence on coverslips, and starved for 24 h in Opti-MEM (Invitrogen) containing 0.2% bovine serum albumin to induce ciliogenesis. Subsequent immunofluorescence analysis was performed as described previously^53,55^. The cells were fixed and permeabilized with 3% paraformaldehyde at 37 °C for 5 min, and subsequently in methanol at − 20 °C for 5 min, and washed three times with phosphate-buffered saline. The fixed/permeabilized cells were blocked with 10% FBS, stained with antibodies diluted in 5% FBS, and observed using an Axio Observer microscope (Carl Zeiss). All images acquired under the same setting and saved in CZI file format were processed and analyzed by using ZEISS ZEN microscope software (Version 3.1; Carl Zeiss). For analysis of ciliary length and the GPR161 staining intensity, a new model of cilia was created by drawing the contour of cilia along the signal of Ac-tubulin or ARL13B in object channel using the Intellesis trainable segmentation module of ZEN. After training many times, the model in the Intellesis trainable segmentation could automatically recognize most cilia. After manually excluding regions that were incorrectly identified as cilia, the Image Analysis application was able to use the model to automeasure ciliary length and the mean fluorescence intensity within cilia. For analysis of the IFT88 staining intensity in the distal region of cilia, a ROI was constructed by using drawing region tools along the ciliary distal region. To correct for local background intensity, the ROI was set to a nearby region. Statistical analyses were performed using GraphPad Prism8 (Version 8.4.3; GraphPad Software, Inc.).

## Supplementary Information

Supplementary Information 1.

Supplementary Information 2.

## Abbreviations

CED: Cranioectodermal dysplasia
EvC: Ellis-van Creveld syndrome
FBS: Fetal bovine serum
GPCR: G protein-coupled receptor
GST: Glutathione *S*-transferase
Hh: Hedgehog
hTERT-RPE1: Human telomerase reverse transcriptase-immortalized retinal pigment epithelial 1
IFT: Intraflagellar transport
JATD: Jeune asphyxiating thoracic dystrophy
KO: Knockout
LIC: Light intermediate chain
mChe: mCherry
Nb: Nanobody
sgRNA: Single-guide RNA
SMO: Smoothened
SRPS: Short rib-polydactyly syndrome
SRTD: Short-rib thoracic dystrophy
TZ: Transition zone
WT: Wild-type
