# Supplementary material for: Combinations of deletion and missense variations of the dynein-2 DYNC2LI1 subunit found in skeletal ciliopathies cause ciliary defects
Source: Sci Rep. 2022 Jan 7;12:31. doi: 10.1038/s41598-021-03950-0 (PMC8742128; doi:10.1038/s41598-021-03950-0)
Supplement: Supplementary file 1 — Supplementary Information 1. [file 41598_2021_3950_MOESM1_ESM.docx]

**Supplemental materials**

**
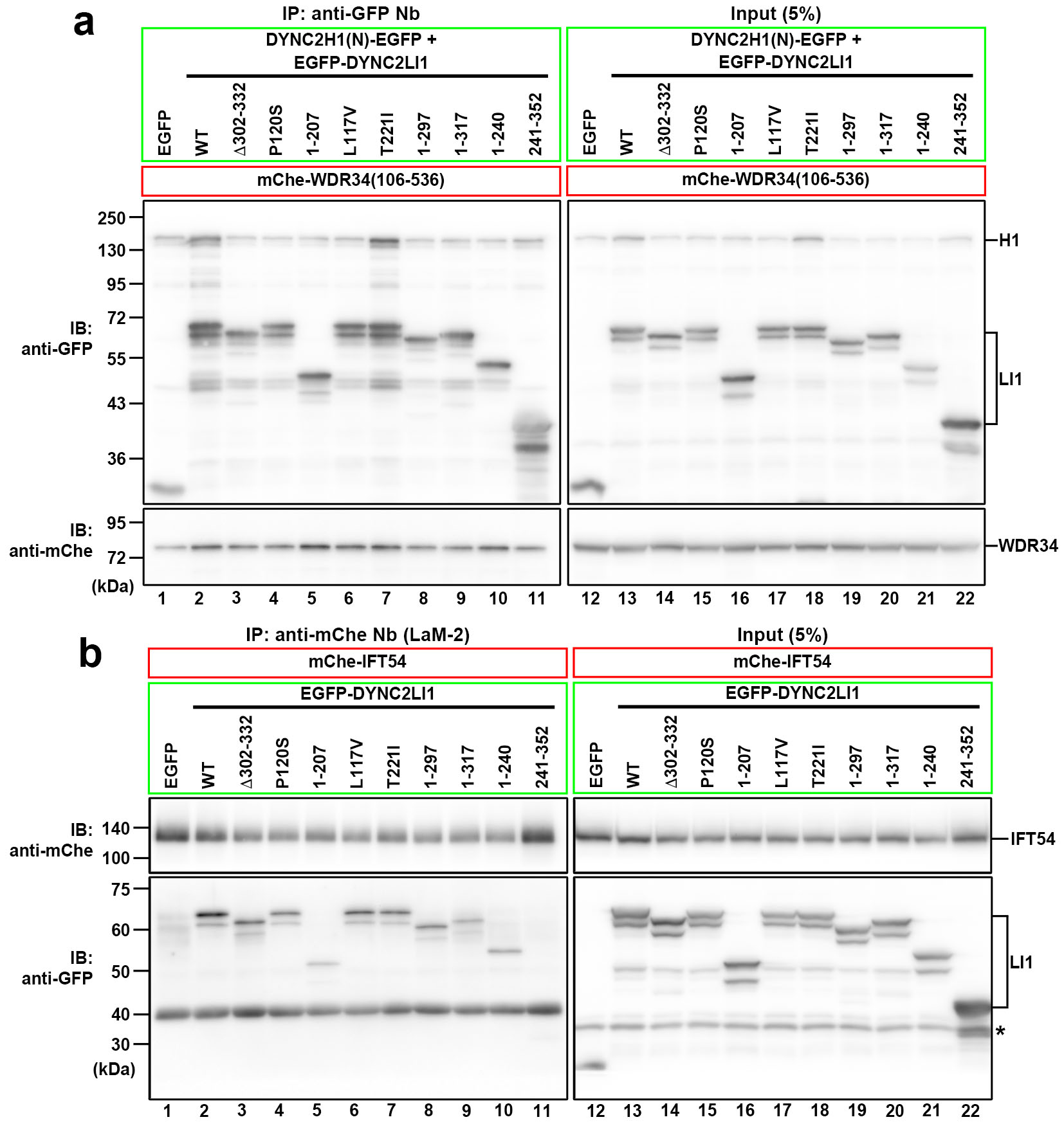
**

**Fig. S1. SRTD variants of DYNC2LI1 are not substantially impaired with respect to interaction with WDR34 or IFT54**

(a) Lysates of cells coexpressing DYNC2H1(N)-EGFP and EGFP-fused DYNC2LI1 constructs, as indicated, together with mChe-WDR34(106–536), were subjected to immunoprecipitation using GST–anti-GFP Nb, followed by immunoblotting analysis using anti-mChe and anti-GFP antibodies. (b) Lysates prepared from HEK293T cells coexpressing EGFP-fused DYNC2LI1 constructs, as indicated, and mChe-IFT54 were subjected to immunoprecipitation using GST-tagged anti-mChe Nb (LaM-2 version), followed by immunoblotting analysis using anti-mChe and anti-GFP antibodies. The asterisk indicates the position of non-specific bands.

**
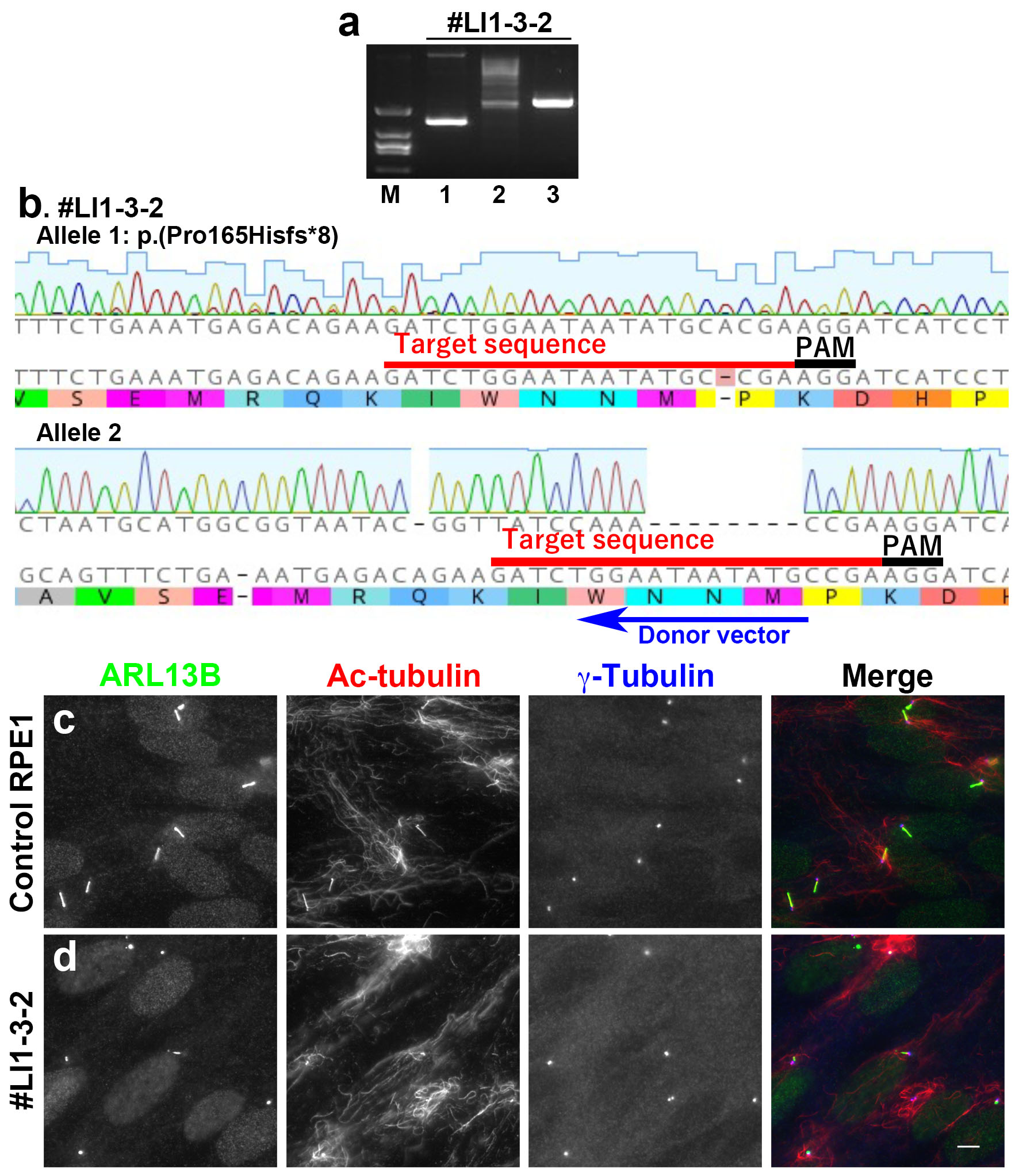
**

**Fig. S2. Characterization of the *DYNC2LI1*-KO cell line**

(a) Genomic DNA extracted from the *DYNC2LI1*-KO cell line #LI1-3-2 was subjected to PCR using the indicated primer pairs (see Table S4) to detect alleles with a small indel or no insertion (lane 1), or with a forward (lane 2) or reverse (lane 3) integration of the donor knockin vector. Lane M, size marker (pSP64 *Dde*I digested). (b) Alignments of allele sequences of the #LI1-3-2 cell line determined by sequencing of the PCR products shown in (A). Red and black lines indicate the target sequence and the protospacer adjacent motif (PAM) sequence, respectively, and the blue arrow indicates the direction of integration of the donor knockin vector. (c, d) Control RPE1 cells (c) and the #LI1-3-2 cell line (d) were serum-starved for 24 h to induce ciliogenesis, and triply immunostained for ARL13B, Ac-tubulin, and γ-tubulin. Scale bar, 5 µm. Note that the #LI1-3-2 cell line has very short cilia demonstrated by staining for ARL13B and Ac-tubulin.

| **Table S1.** **Pathogenic *DYNC2LI1* variants identified in patients with skeletal ciliopathies** | | | | |
| --- | --- | --- | --- | --- |
|  | DNA variation | Location | Predicted amino acid change | Reference |
| R01-013A | c.349C>G  c.996+1G>A | Exon 6  Intron 12 | p.(Leu117Val)  p.(Ser302_Ile332del) | Taylor et al. (2015) |
| R07-628A | c.349C>G  c.372G>A | Exon 6  Exon 6 | p.(Leu117Val)  p.(Trp124*) | Taylor et al. (2015) |
| R03-303A | c.996+3A>G  c.1003G>T | Intron 12  Exon 13 | p.(Ser302_Ile332del)  p.(Glu335*) | Taylor et al. (2015) |
| One family | c.622C>T  c.662C>T | Exon 8  Exon 9 | p.(Arg208*)  p.(Thr221Ile) | Kessler et al. (2015) |
| Family 1 | c.2T>C  c.662C>T | Exon 1  Exon 9 | p.(Met1?)  p.(Thr221Ile) | Niceta et al. (2018) |
| Family 2 | c.462delA  c.662C>T | Exon 6  Exon 9 | p.(Val141*)  p.(Thr221Ile) | Niceta et al. (2018) |
| Family 3 | c.123_124insA  c.658-11delT | Exon 2  Intron 8 | p.(Gly42Argfs12*)  p.? | Niceta et al. (2018) |
| One family | c.358C>T  c.928A>T | Exon 6  Exon 12 | p.(Pro120Ser)  p.(Lys310*) | Zhang et al. (2020) |

| **Table S2. Plasmids used in this study** | |  |
| --- | --- | --- |
| Vector | Insert | Reference |
| pCAG2-EGFP-C | DYNC2LI1 | Hamada et al., 2018 |
| pCAG2- mCherry-C | WDR34(106–536) | Tsurumi et al., 2019 |
| pCAG2-EGFP-N | DYNC2H1(N; 1–1,090) | This study |
| pCAG2-mCherry-N | DYNC2H1(N; 1–1,090) | This study |
| pCAG2- mCherry-C | WDR60(627–1,066) | This study |
| pCAG2- mCherry-C | IFT54 | This study |
| pCAG2-EGFP-C | DYNC2LI1(Δ302–332) | This study |
| pCAG2-EGFP-C | DYNC2LI1(P120S) | This study |
| pCAG2-EGFP-C | DYNC2LI1(1-207) | This study |
| pCAG2-EGFP-C | DYNC2LI1(L117V) | This study |
| pCAG2-EGFP-C | DYNC2LI1(T221I) | This study |
| pCAG2-EGFP-C | DYNC2LI1(1–297) | This study |
| pCAG2-EGFP-C | DYNC2LI1(1–317) | This study |
| pCAG2-EGFP-C | DYNC2LI1(1–240) | This study |
| pCAG2-EGFP-C | DYNC2LI1(241–352) | This study |
| pRRLsinPPT-mCherry-C-IRES-Zeo | DYNC2LI1 | Hamada et al., 2018 |
| pRRLsinPPT-mCherry-C-IRES-Zeo | DYNC2LI1(Δ302–332) | This study |
| pRRLsinPPT-mCherry-C-IRES-Zeo | DYNC2LI1(P120S) | This study |
| pRRLsinPPT-mCherry-C-IRES-Zeo | DYNC2LI1(1–207) | This study |
| pRRLsinPPT-mCherry-C-IRES-Zeo | DYNC2LI1(L117V) | This study |
| pRRLsinPPT-mCherry-C-IRES-Zeo | DYNC2LI1(T221I) | This study |
| pRRLsinPPT-mCherry-C-IRES-Zeo | DYNC2LI1(1–297) | This study |
| pRRLsinPPT-mCherry-C-IRES-Zeo | DYNC2LI1(1–317) | This study |
| pRRLsinPPT-mCherry-C-IRES-Zeo | DYNC2LI1(1–240) | This study |
| pDonor-tBFP-NLS-Neo (Universal) | − | Katoh et al., 2017 |
| peSpCas9 (1.1)-2×gRNA | − | Katoh et al., 2017 |
| pGEX-6P1 | Anti-GFP-nanobody | Katoh et al., 2015 |
| pGEX-6P1 | Anti-mCherry-Nanobody (LaM-2) | Ishida et al., 2021 |

| **Table S3. Antibodies used in this study** | | |  | |  | |
| --- | --- | --- | --- | --- | --- | --- |
| Antibody | Manufacturer | Clone/catalog number or reference number | | Dilution (purpose) | |  |
| Polyclonal rabbit anti-IFT88 | Proteintech | 13967-1-AP | | 1:500 (IF) | |  |
| Polyclonal rabbit anti-GPR161 | Proteintech | 13398-1-AP | | 1:500 (IF) | |  |
| Polyclonal rabbit anti-ARL13B | Proteintech | 17711-1-AP | | 1:500 (IF) | |  |
| Monoclonal mouse anti-ARL13B | Abcam | N295B/66 | | 1:500 (IF) | |  |
| Monoclonal mouse anti-FOP | Abnova | 2B1 | | 1:10,000 (IF) | |  |
| Monoclonal mouse anti-Ac-α-tubulin | Sigma-Aldrich | 6-11B-1 | | 1:1,000 (IF) | |  |
| Monoclonal mouse anti-γ-tubulin | Sigma-Aldrich | GTU88 | | 1:1,000 (IF) | |  |
| Monoclonal mouse anti-RFP | MBL | 3G5 | | 1:1,000 (IF) | |  |
| Polyclonal rabbit anti-mCherry | Proteintech | 26765-1-AP | | 1:10,000 (IB) | |  |
| Monoclonal mouse anti-GFP | Proteintech | 66002-1-Ig | | 1:10,000 (IB) | |  |
| Monoclonal mouse anti-GAPDH | Ambion | 6C5 | | 1:10,000 (IB) | |  |
| AlexaFluor-conjugated secondary | Molecular Probes | A11034, A27039, A21244,  A11004, A21127, A21240, A21241, A21131, A21242 | | 1:1,000 (IF) | |  |
| Peroxidase-conjugated secondary | Jackson ImmunoResearch | 115-035-166, 111-035-144 | | 1:3,000 (IB) | |  |

IF, immunofluorescence; IB, immunoblotting

| **Table S4. Oligo DNAs used in this study** | |
| --- | --- |
| Name | Sequence |
| pTagBFP-N-RV2 (primer 3) | 5'-CGTAGAGGAAGCTAGTAGCCAGG-3' |
| *DYNC2LI1*-genome#1-FW (primer 1) | 5'- GGTCATCTGGTTAAAGTGTTGAAGT-3' |
| *DYNC2LI1*-genome#1-RV (primer 2) | 5'- GGGCTGTGGTCACCCCAGAC-3' |
| *DYNC2LI1*-genome#2-FW (primer 4) | 5'- TGATGCATTTTGAGGGATGGGA-3' |
| *DYNC2LI1*-genome#2-RV (primer 5) | 5'- TATCTCAGTTCAGCACGGGC-3' |
| *DYNC2LI1*-genome#1-FW (primer 6) | 5'- TCGATGCAAATGCTTGGAGC-3' |
| *DYNC2LI1*-genome#1-FW (primer 7) | 5'- CCTTGTTTTGCTCTTACTGAGGT-3' |
| *DYNC2LI1*-gRNA#1-S | 5'- CACCGCTTCATTGGCAGTAAAAATG-3' |
| *DYNC2LI1*-gRNA#1-AS | 5'- AAACCATTTTTACTGCCAATGAAGC-3' |
| *DYNC2LI1*-gRNA#2-S | 5'- CACCGCTCACTTTTGGGAACTCGG-3' |
| *DYNC2LI1*-gRNA#2-AS | 5'- AAACCCGAGTTCCCAAAAGTGAGC-3' |
| *DYNC2LI1*-gRNA#3-S | 5'- CACCGATCTGGAATAATATGCCGA-3' |
| *DYNC2LI1*-gRNA#3-AS | 5'- AAACTCGGCATATTATTCCAGATC-3' |
| SLiCE-EcoRI-*DYNC2LI1*-FW | 5'- CTCGAGCTCAAGCTTCGAATTCTATGCCCAGTGAAACTCTCTGGG-3' |
| SLiCE-SalI-*DYNC2LI1*-RV | 5'- CCGGGCCCGCGGTACCGTCGACTCAAGAATCAAGCTCGATTTGTTTC-3' |
| SLiCE-pRRL-DYNC2LI1-S | 5'- AAGTCCGGCCGGACTCAGGTTATGCCCAGTGAAACTCTCTG-3' |
| SLiCE-pRRL-*DYNC2LI1*-AS | 5'- TCCAGCACACTGGATCACTCGACTCAAGAATCAAGCTC-3' |
| SLICE-SalI-*DYNC2LI1*-240aa-RV | 5'- CCCGGGCCCGCGGTACCGTCGACTCAAAATGCCAACTGGTTGATAACTCC-3' |
| SLICE-EcoRI-*DYNC2LI1*-241aa-FW | 5'- TCTCGAGCTCAAGCTTCGAATTCTGGCATTGACAAAAGCAAATCAATA-3' |
| *DYNC2LI1*-L117V-FW | 5'- GTTCTGGATGTTTCAAAACCTAATGATCTC-3' |
| *DYNC2LI1*-L117V-RV | 5'- GTTTTGAAACATCCAGAACGAGAACAAGAG-3' |
| *DYNC2LI1*-T221I-FW | 5'- AATGTTTATCAGTAAATCAGAAGCTCTATTAC-3' |
| *DYNC2LI1*-T221I-RV | 5'- GATTTACTGATAAACATTAATGATGCTCC-3' |
| SLiCE-SalI-*DYNC2LI1*-297aa-RV | 5'- CCGGGCCCGCGGTACCGTCGACTCAGAGCTTTTCATACACTTTTTTCC-3' |
| SLiCE-SalI-*DYNC2LI1*-317aa-RV | 5'- CCGGGCCCGCGGTACCGTCGACTCACTGAGGATCTCTCGCAGGGTCC-3' |
| SLiCE-DYNC2LI1-△Exon12-FW | 5'- AGTGTATGAAAAGCTCTTTCCACCAAAGGAACTGGAACAG-3' |
| SLiCE-*DYNC2LI1*-△Exon12-RV | 5'- GTTCCAGTTCCTTTGGTGGAAAGAGCTTTTCATACACTTT-3' |
| SLiCE-*DYNC2LI1*-P120S-FW | 5'- CTTTCAAAATCTAATGATCTCTGGCCCACCATGGA-3' |
| SLiCE-*DYNC2LI1*-P120S-RV | 5'- GAGATCATTAGATTTTGAAAGATCCAGAACGAGAACAAGAG-3' |
| SLiCE-*DYNC2LI1*-R208X-FW | 5'- AAGACACTTTGATTTGTTGCACATTATTATGGAGC-3' |
| SLiCE-*DYNC2LI1*-R208X-RV | 5'- GCAACAAATCAAAGTGTCTTGCATATTACCTTTCTCTTCT-3' |
| SLiCE-pRRL-*DYNC2LI1*-240aa-AS | 5'- CCAGCACACTGGATCACTCGACTCAAAATGCCAACTGGTTGATAACTCC-3' |
| SLiCE-pRRL-*DYNC2LI1*-2970aa-AS | 5'- CCAGCACACTGGATCACTCGACTCAGAGCTTTTCATACACTTTTTTCC-3' |
| SLiCE-pRRL-*DYNC2LI1*-317aa-AS | 5'- CCAGCACACTGGATCACTCGACTCACTGAGGATCTCTCGCAGGGTCC-3' |

FW, forward; RV, reverse; S, sense; AS, antisense

**Supplemental references**

Hamada, Y., Tsurumi, Y., Nozaki, S., Katoh, Y. & Nakayama, K. Interaction of WDR60 intermediate chain with TCTEX1D2 light chain of the dynein-2 complex is crucial for ciliary protein trafficking. *Mol. Biol. Cell* **29**, 1628-1639 (2018).

Ishida, Y., Kobayashi, T., Chiba, S., Katoh, Y. & Nakayama, K. Molecular basis of ciliary defects caused by compound heterozygous *IFT144/WDR19* mutations found in cranioectodermal dysplasia. *Hum. Mol. Genet.* **30**, 213-225 (2021).

Katoh, Y. *et al.* Practical method for targeted disruption of cilia-related genes by using CRISPR/Cas9-mediated homology-independent knock-in system. *Mol. Biol. Cell* **28**, 898-906 (2017).

Katoh, Y., Nozaki, S., Hartanto, D., Miyano, R. & Nakayama, K. Architectures of multisubunit complexes revealed by a visible immunoprecipitation assay using fluorescent fusion proteins. *J. Cell Sci.* **128**, 2351-2362 (2015).

Kessler, K. *et al.* *DYNC2LI1* mutations broaden the clinical spectrum of dynein-2 defects. *Sci. Rep.* **5**, 11649 (2015).

Niceta, M. *et al.* Biallelic mutations in DYNC2LI1 are a rare cause of Ellis-van Creveld syndrome. *Clin. Genet.* **93**, 632-639 (2018).

Taylor, S. P. *et al.* Mutations in *DYNC2LI1* disrupt cilia function and cause short rib polydactyly syndrome. *Nat. Commun.* **6**, 7092 (2015).

Tsurumi, Y., Hamada, Y., Katoh, Y. & Nakayama, K. Interactions of the dynein-2 intermediate chain WDR34 with the light chains are required for ciliary retrograde protein trafficking. *Mol. Biol. Cell* **30**, 658-670 (2019).

Zhang, X. *et al.* Whole-exome sequencing identified two novel mutations of *DYNC2LI1* in fetal skeletal ciliopathy. *Mol. Genet. Genomic Med.* **8**, e1524 (2020).
