# Supplementary material for: Combinations of deletion and missense variations of the dynein-2 DYNC2LI1 subunit found in skeletal ciliopathies cause ciliary defects
Source: Sci Rep. 2022 Jan 7;12:31. doi: 10.1038/s41598-021-03950-0 (PMC8742128; doi:10.1038/s41598-021-03950-0)
Supplement: Supplementary file 2 — Supplementary Information 2. [file 41598_2021_3950_MOESM2_ESM.pptx]

## Slide 1
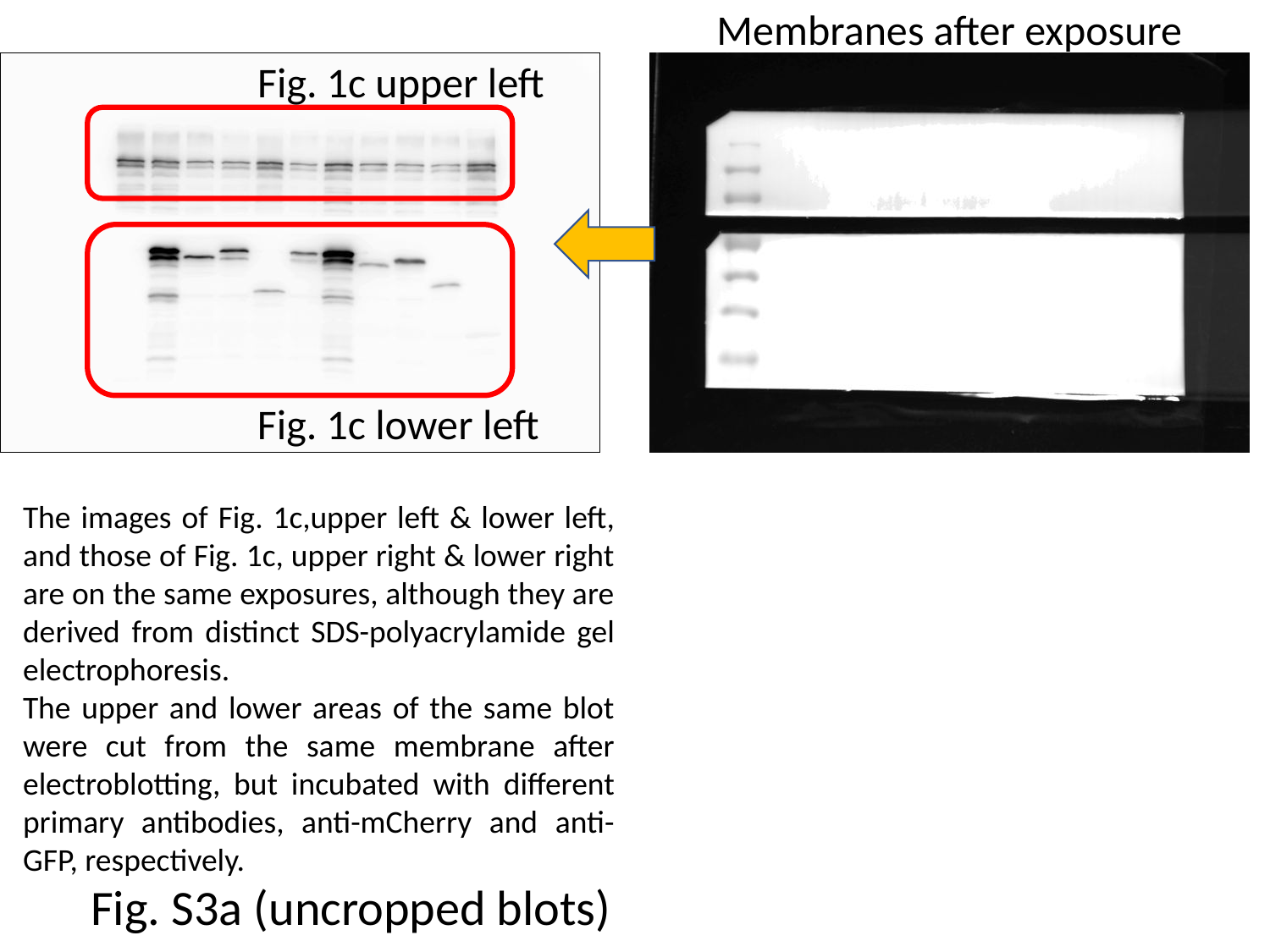

Membranes after exposure
Fig. 1c upper left
Fig. 1c lower left
The images of Fig. 1c,upper left & lower left, and those of Fig. 1c, upper right & lower right are on the same exposures, although they are derived from distinct SDS-polyacrylamide gel electrophoresis.
The upper and lower areas of the same blot were cut from the same membrane after electroblotting, but incubated with different primary antibodies, anti-mCherry and anti-GFP, respectively.
Fig. S3a (uncropped blots)

## Slide 2
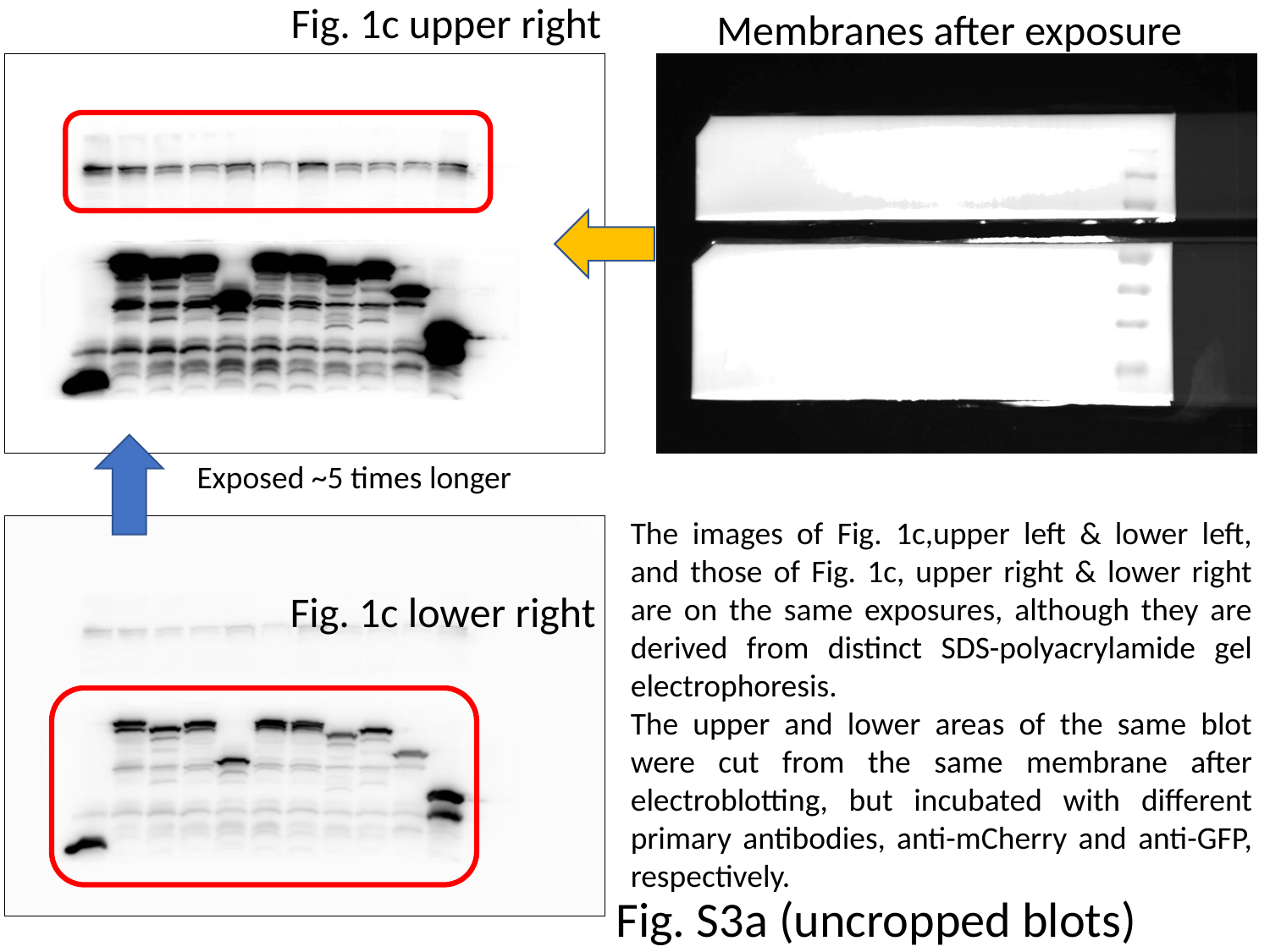

Fig. 1c upper right
Membranes after exposure
Exposed ~5 times longer
The images of Fig. 1c,upper left & lower left, and those of Fig. 1c, upper right & lower right are on the same exposures, although they are derived from distinct SDS-polyacrylamide gel electrophoresis.
The upper and lower areas of the same blot were cut from the same membrane after electroblotting, but incubated with different primary antibodies, anti-mCherry and anti-GFP, respectively.
Fig. 1c lower right
Fig. S3a (uncropped blots)

## Slide 3
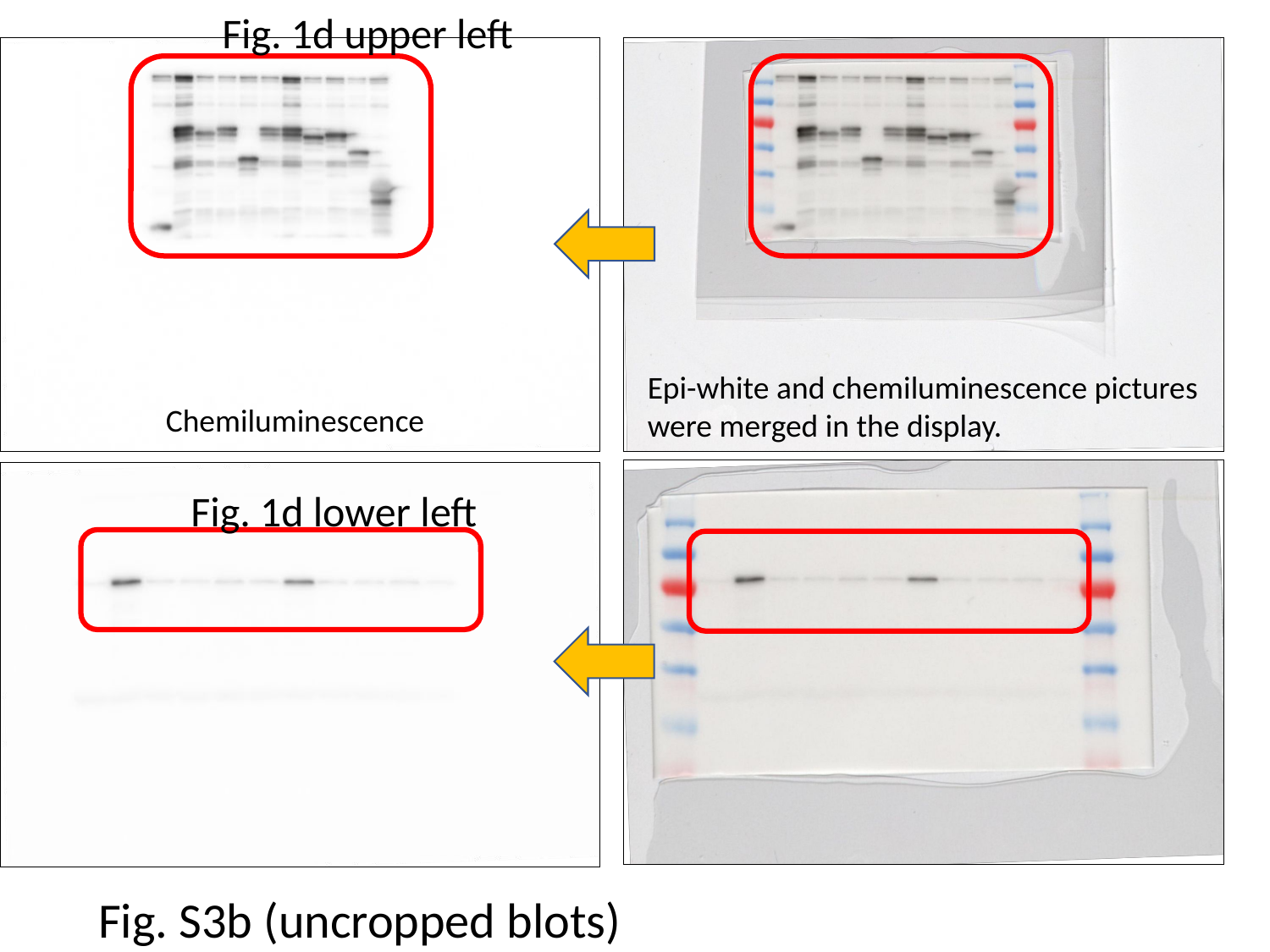

Fig. 1d upper left
Epi-white and chemiluminescence pictures were merged in the display.
Chemiluminescence
Fig. 1d lower left
Fig. S3b (uncropped blots)

## Slide 4
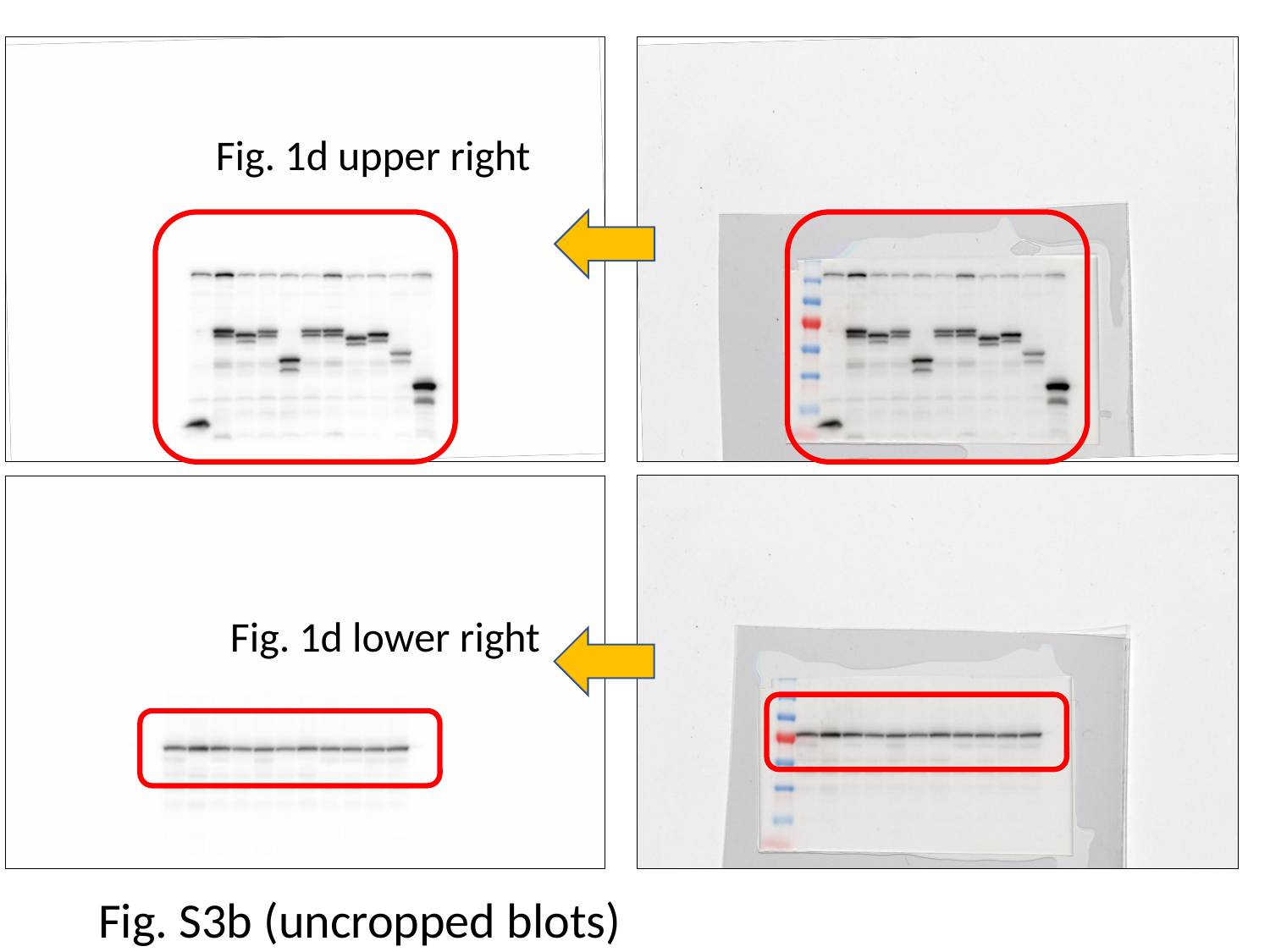

Fig. 1d upper right
Fig. 1d lower right
Fig. S3b (uncropped blots)

## Slide 5
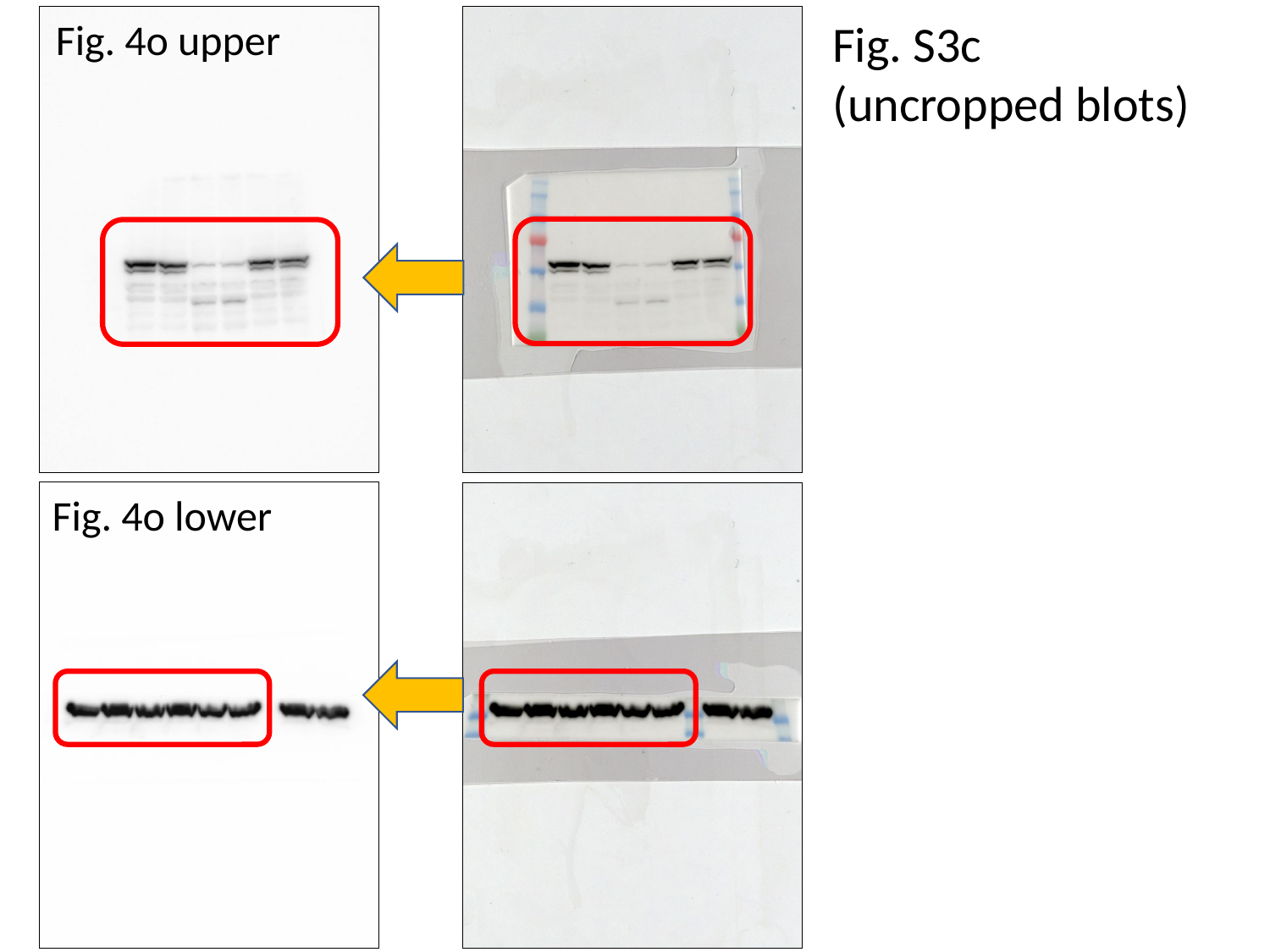

Fig. 4o upper
Fig. S3c
(uncropped blots)
Fig. 4o lower

## Slide 6
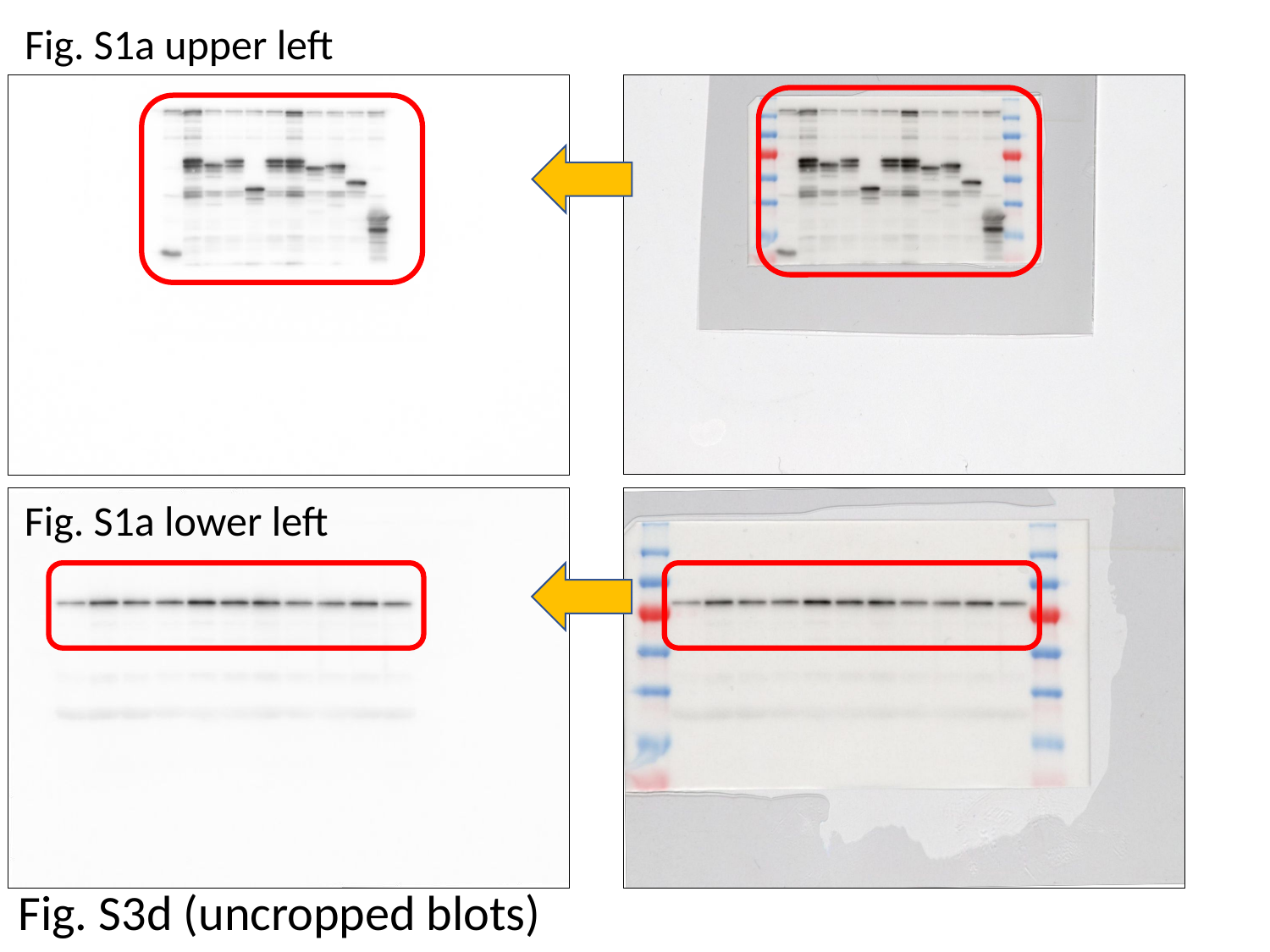

Fig. S1a upper left
Fig. S1a lower left
Fig. S3d (uncropped blots)

## Slide 7
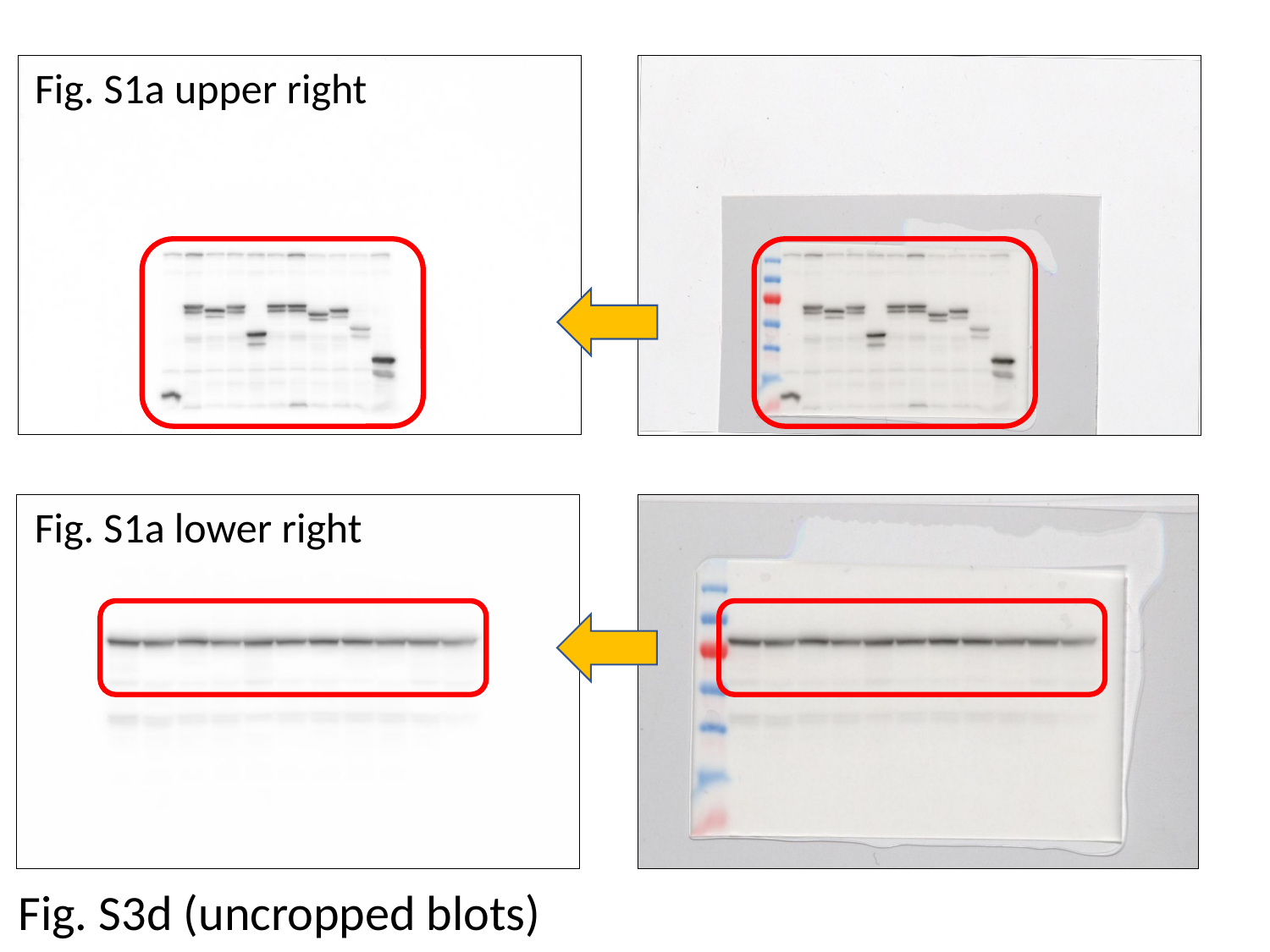

Fig. S1a upper right
Fig. S1a lower right
Fig. S3d (uncropped blots)

## Slide 8
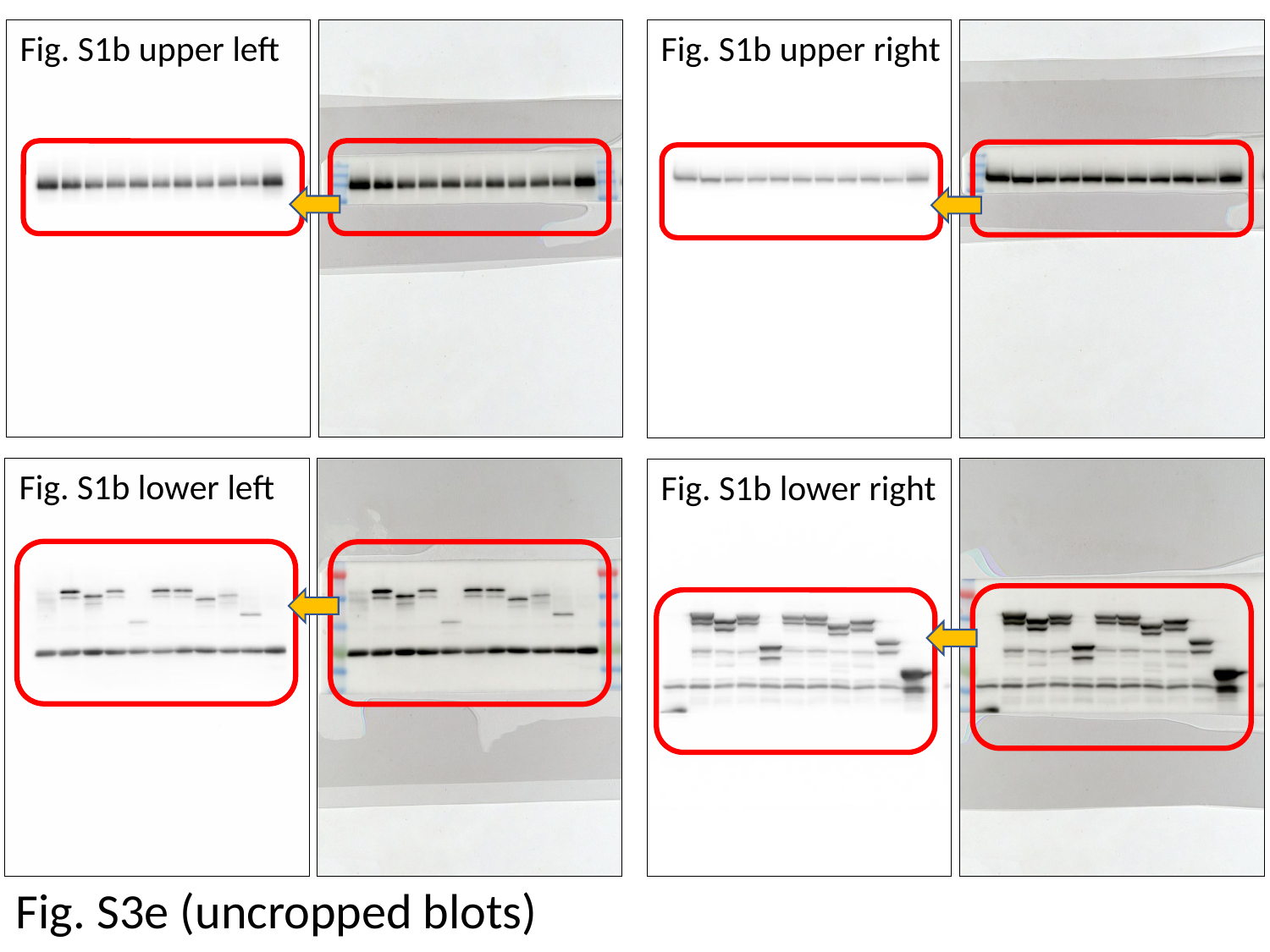

Fig. S1b upper left
Fig. S1b upper right
Fig. S1b lower left
Fig. S1b lower right
Fig. S3e (uncropped blots)

## Slide 9
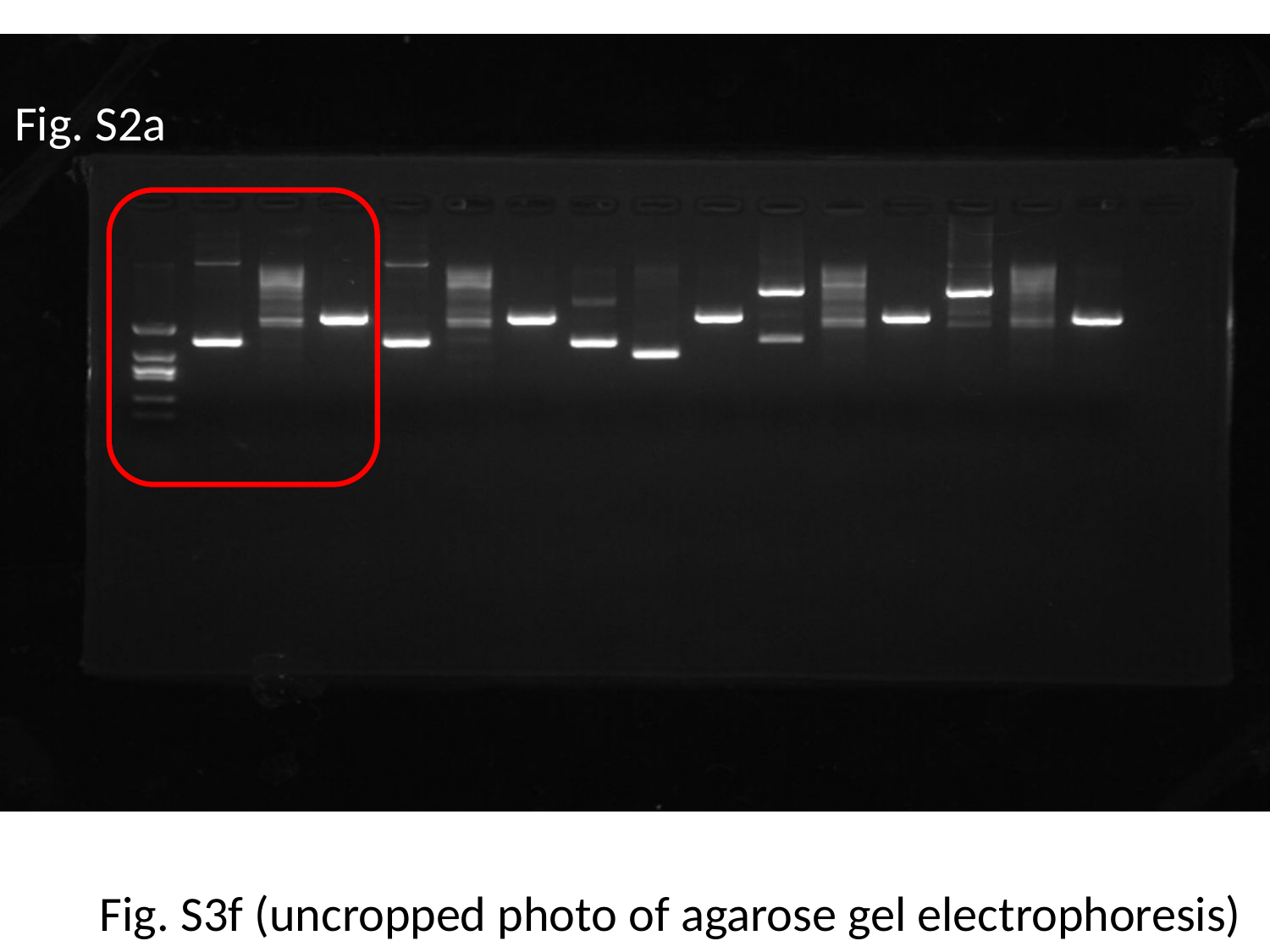

Fig. S2a
Fig. S3f (uncropped photo of agarose gel electrophoresis)
